# Recombinant Paraprobiotics as a New Paradigm for Treating Gastrointestinal Nematode Parasites of Humans

**DOI:** 10.1128/AAC.01469-20

**Published:** 2021-02-17

**Authors:** Hanchen Li, Ambily Abraham, David Gazzola, Yan Hu, Gillian Beamer, Kelly Flanagan, Ernesto Soto, Florentina Rus, Zeynep Mirza, Austin Draper, Sridhar Vakalapudi, Cheryl Stockman, Perry Bain, Joseph F. Urban, Gary R. Ostroff, Raffi V. Aroian

**Affiliations:** a Program in Molecular Medicine, University of Massachusetts Medical School, Worcester, Massachusetts, USA; b Department of Infectious Disease and Global Health, Tufts University, North Grafton, Massachusetts, USA; c Synthetic Biomanufacturing Facility, Utah State University, Logan, Utah, USA; d Cummings School of Veterinary Medicine at Tufts University, North Grafton, Massachusetts, USA; e United States Department of Agriculture, Agricultural Research Service, Beltsville Human Nutrition Research Center, Diet, Genomics, Immunology Laboratory, Beltsville, Maryland, USA

**Keywords:** *Bacillus thuringiensis*, anthelmintic, crystal protein, gastrointestinal nematodes, helminths, hookworms, paraprobiotic, soil-transmitted helminths

## Abstract

Gastrointestinal nematodes (GINs) of humans, e.g., hookworms, negatively impact childhood growth, cognition, nutrition, educational attainment, income, productivity, and pregnancy. Hundreds of millions of people are targeted with mass drug administration (MDA) of donated benzimidazole anthelmintics. However, benzimidazole efficacy against GINs is suboptimal, and reduced/low efficacy has been seen. Developing an anthelmintic for human MDA is daunting: it must be safe, effective, inexpensive, stable without a cold chain, and massively scalable. Bacillus thuringiensis crystal protein 5B (Cry5B) has anthelmintic properties that could fill this void. Here, we developed an active pharmaceutical ingredient (API) containing B. thuringiensis Cry5B compatible with MDA. We expressed Cry5B in asporogenous B. thuringiensis during vegetative phase, forming cytosolic crystals. These bacteria with cytosolic crystals (BaCC) were rendered inviable (inactivated BaCC [IBaCC]) with food-grade essential oils. IBaCC potency was validated *in vitro* against nematodes. IBaCC was also potent *in vivo* against human hookworm infections in hamsters. IBaCC production was successfully scaled to 350 liters at a contract manufacturing facility. A simple fit-for-purpose formulation to protect against stomach digestion and powdered IBaCC were successfully made and used against GINs in hamsters and mice. A pilot histopathology study and blood chemistry workup showed that five daily consecutive doses of 200 mg/kg body weight Cry5B IBaCC (the curative single dose is 40 mg/kg) was nontoxic to hamsters and completely safe. IBaCC is a safe, inexpensive, highly effective, easy-to-manufacture, and scalable anthelmintic that is practical for MDA and represents a new paradigm for treating human GINs.

## Abstract (precis)

Gastrointestinal nematodes (GINs) of humans, e.g., hookworms, negatively impact childhood growth, cognition, nutrition, educational attainment, income, productivity, and pregnancy. Hundreds of millions of people are targeted with mass drug administration (MDA) of donated benzimidazole anthelmintics.

## INTRODUCTION

Among the neglected tropical diseases, soil-transmitted helminths/nematodes (STHs/STNs) or gastrointestinal nematodes (GINs) collectively affect the largest number of people, with a global estimate of >1.5 billion infected individuals (1). Human GINs include hookworms (Ancylostoma duodenale, Ancylostoma ceylanicum, and Necator americanus), ascarids (Ascaris lumbricoides), and whipworms (Trichuris trichiura) (2). Human GIN parasites have an enormous impact on children, leading to physical growth stunting, cognitive impairment, malnutrition, anemia, impaired physical fitness, loss of future income, decreased educational attainment, and defective immune responses to other infectious diseases (e.g., HIV, malaria, and tuberculosis) and vaccines (1–3). These parasites also cause significant complications for pregnant women and significant reductions in adult worker productivity, accounting for >5 million disability-adjusted life years and productivity losses of more than $100 billion annually, with the majority of the morbidity attributed to hookworms (2, 4–7).

Only one class of drug, the benzimidazoles, is approved and suitable for single-dose mass drug administration (8, 9). GIN resistance to these drugs develops readily and is extremely common in veterinary medicine, where they have been used much longer and more intensely than in human medicine (8, 10). Against human GINs, these drugs have poor efficacy against whipworms. Low efficacy of albendazole (the most efficacious benzimidazole used in humans) against hookworms and *Ascaris* has been reported in many locales, with definitive benzimidazole resistance alleles detected in natural populations of human hookworms in Kenya and Brazil (11–20).

New mechanism-of-action anthelmintics are urgently needed in the pipeline, as the lead time for drugs to reach the market is years. As human GIN mass drug administration intensifies, further loss of benzimidazole efficacy is highly likely. However, developing new drugs for humans GINs is exceedingly difficult: (i) drug development is very expensive, costing several billions (21), a cost that cannot easily be recouped for diseases of the poorest peoples; (ii) the number of people impacted is enormous—any new therapy has to be massively scalable (an estimated 1.5 billion doses currently needed for children and women alone) and inexpensive (benzimidazoles are currently donated) (22); (iii) the therapy has to withstand harsh environmental conditions without a cold chain; and (iv) the therapy has to be safe and effective. Thus, the normal rules of “market-incentive” drug development do not apply. In fact, no drug has ever been developed for human GINs; all drugs used are expanded label uses of drugs developed for veterinary medicine. Of the two drugs used in mass drug administration today, albendazole was approved in humans in 1982 (23) and mebendazole was approved in 1974 (USA [24]). No new drugs have thus entered human GIN treatment for more than 30 years.

Bacillus thuringiensis crystal (Cry) proteins have the potential to offer a novel, natural, safe, and broad-spectrum anthelmintic alternative. B. thuringiensis spores are mass produced globally as a biopesticide, encompassing ∼75% of the bioinsecticide market (25). The main insecticidal components of B. thuringiensis are three-domain (3D)-Cry proteins that bind specifically to the invertebrate intestine, damaging the gut and killing the invertebrate target (26). 3D-Cry proteins have also been engineered into a range of food crops and are expressed in ∼100 mHa of transgenic crops worldwide (27). More than a dozen different 3D-Cry proteins have been tested and found to be completely safe to vertebrates at doses of ≫1,000 mg/kg of body weight and are FDA/EPA approved for ingestion (28, 29). The 3D-Cry protein Cry5B is related to the 3D-Cry proteins used as insecticides but targets nematodes instead. When administered orally as spore crystal lysates (SCLs) (a mix of B. thuringiensis spores and Cry protein crystals as they naturally occur), Cry5B is highly effective against GIN infections in hamsters, pigs, and dogs (30–34). The nematode receptor for Cry5B is an invertebrate-specific glycolipid absent in vertebrates (35).

Developing 3D-Cry proteins as large-scale ingested therapeutics compatible with human mass drug administration, however, requires a very different set of considerations than application as topical or transgenic biopesticides and insecticides. Here, we address these considerations, developing an active pharmaceutical ingredient (API) based on Cry5B compatible with safety, cost, scale, ease of production, and stability required for human mass drug administration.

## RESULTS

### Inactivated bacteria with cytosolic crystals.

We reasoned that delivering Cry5B in a live bacterium (B. thuringiensis or otherwise) to humans would be problematic given that (i) B. thuringiensis is closely related to Bacillus cereus, which can cause food poisoning, (ii) release of live recombinant bacteria has environmental concerns, because live bacteria in the soil could select for Cry5B resistance against free-living stages of hookworms, (iii) live bacteria could replicate in the human gastrointestinal tract (amplifying environmental and resistance concerns), (iv) there are uncertainties and significant safety concerns in the responses of billions of people to live bacteria, (v) degradation of live bacteria during storage could reduce potency and stability, and (vi) delivery of a stable live bacterial therapeutic around the world for mass drug administration would be difficult to achieve (36–40). Producing and delivering Cry5B as a purified protein is also problematic, as making enough purified protein cheaply and massively for mass drug administration is difficult to envision.

**FIG 1:**
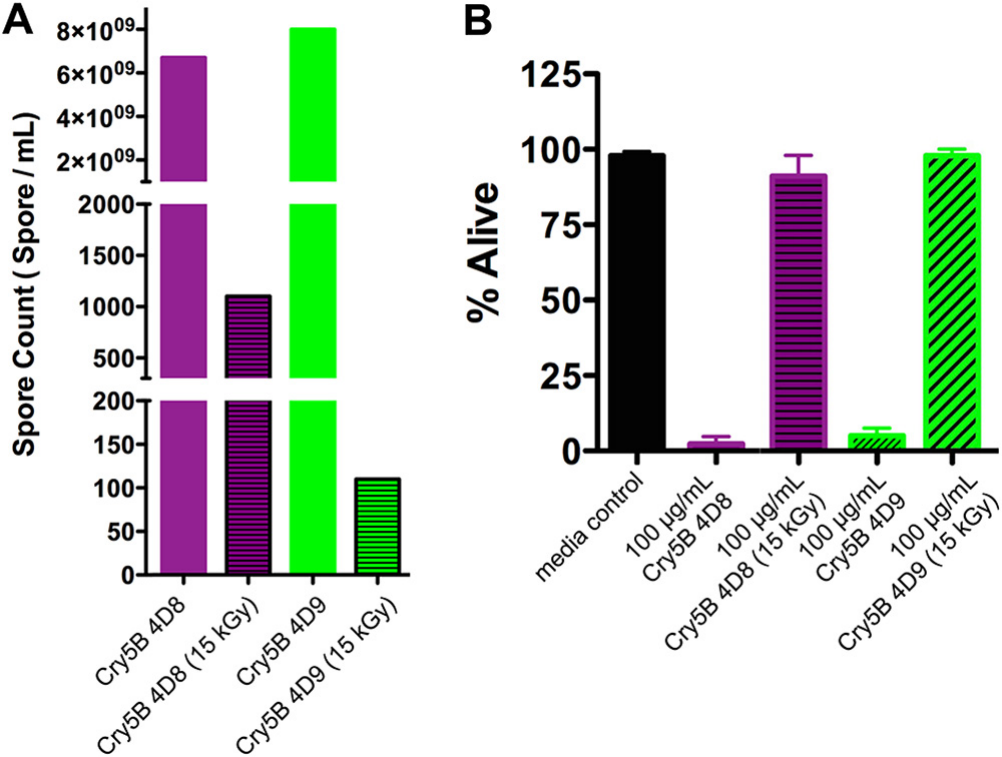
Effects of gamma irradiation on B. thuringiensis spore viability and Cry5B bioactivity. (A) Effect of 15 kGy of gamma irradiation on spore counts for two different Cry-deficient B. thuringiensis strains (4.D.8 and 4.D.9) transformed with a Cry5B-expressing plasmid. (B) Comparison of Cry5B efficacy expressed in 4.D.8 and 4.D.9 on C. elegans viability at 100 µg/ml before and after 15-kGy irradiation. Labels without “15 kGy” indicate non-gamma-irradiated samples.

We therefore set out to deliver Cry5B, without purification of the protein, as part of a dead bacterial product (paraprobiotic [39]). To make a Cry5B paraprobiotic, we initially turned to and produced Cry5B spore crystal lysates, which have been used for most published *in vivo* studies of Cry5B anthelmintic efficacy as well as all B. thuringiensis insecticidal production and use (31, 33, 34, 41, 42). We attempted to inactivate (kill) the spores without losing activity of the crystal protein using gamma irradiation (43). Although gamma irradiation resulted in a significant (10^6^ to 10^7^) reduction in spore viability (Fig. 1A), the procedure completely killed Cry5B antinematode activity (Fig. 1B). Preliminary studies using chemical treatment instead of gamma irradiation to inactivate spores without damaging crystal activity yielded similar, disappointing results.

**FIG 2:**
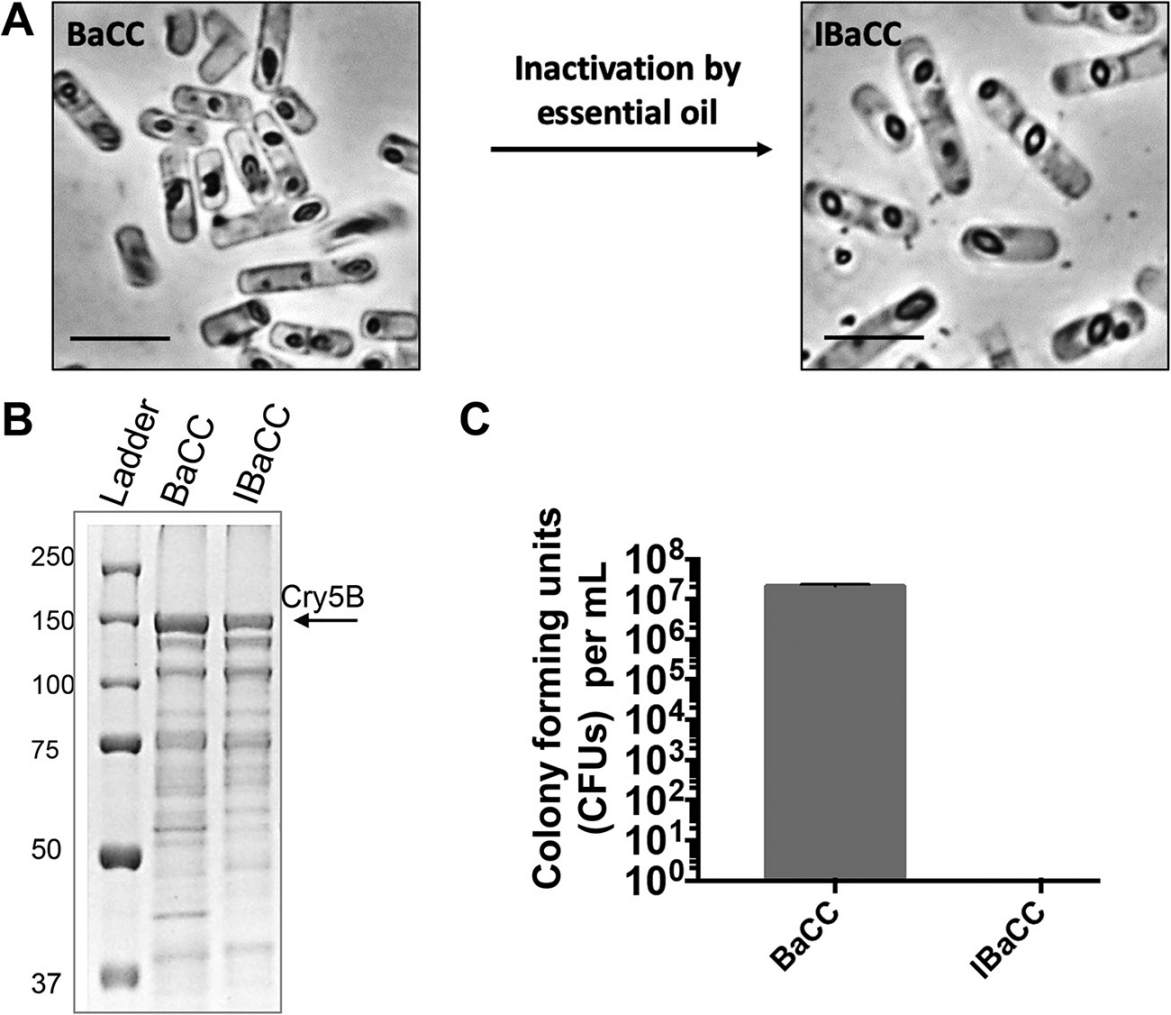
BaCC (bacteria with cytosolic crystals) and IBaCC (Inactivated bacteria with cytosolic crystals). (A) spo0A-deficient B. thuringiensis cells expressing Cry5B from a vegetative promoter before (BaCC) and after (IBaCC) treatment with essential oil. Cry5B bipyramidal crystals (dark) are evident inside the bacteria pre- and posttreatment. Scale bars, 5 µm. (B) Protein gel showing Cry5B protein expressed in spo0A-deficient cells before and after essential oil treatment. (C) Spore counts from spo0A-deficient cells expressing Cry5B before and after essential oil treatment along with standard deviation (actual spore counts, 2.1 × 10^7^ CFU/ml in BaCC and 0 CFU/ml in IBaCC).

We therefore decided that a better approach would be to express Cry5B in vegetative bacteria that could be more easily killed (inactivated). Based on our previous experiences and based on the fact that the inactivation process needs to be compatible with human ingestion, we hypothesized that food-grade monoterpenes derived from essential oils would be effective and safe antimicrobials for this application (44–47). We therefore expressed Cry5B in asporogenous (spo0A-deficient) B. thuringiensis so that the Cry5B crystals would be formed inside a vegetative B. thuringiensis without subsequent spore formation (Fig. 2A) (48, 49). We call such cells BaCC, for bacteria with cytosolic crystals. A number of food-grade monoterpenes were found that were capable of inactivation (killing) spo0A-deficient cells (see Table S1 in the supplemental material). When Cry5B was expressed in these cells and inactivated with terpene, the crystals stayed trapped within the cells, and the protein remained intact (Fig. 2A and B). A >10^7^-fold reduction in CFU was seen upon terpene treatment, with often no viable cells detected (Fig. 2C). We call these cells IBaCC, for inactivated BaCC.

### IBaCC is an active nematicide.

**FIG 3:**
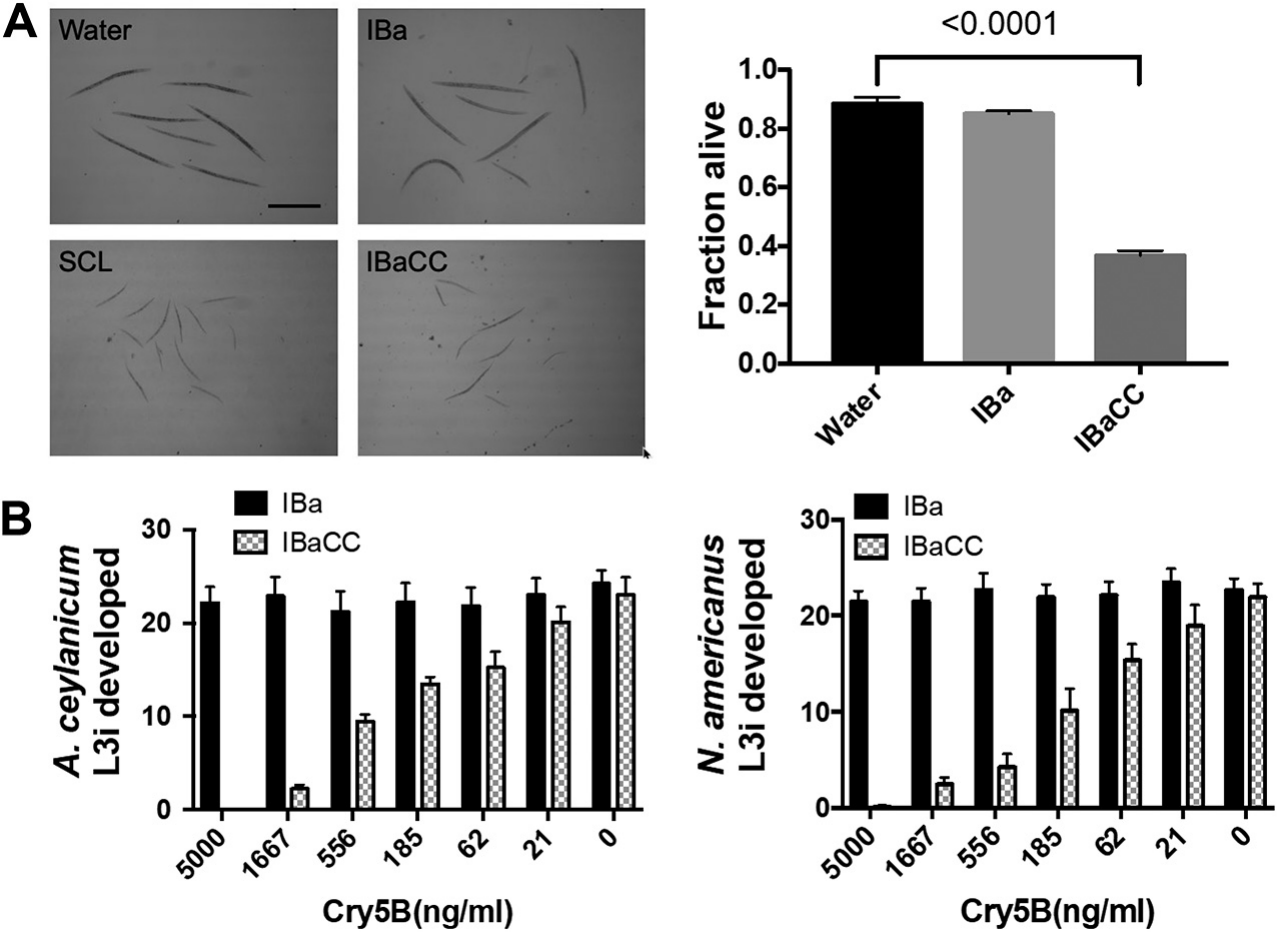
Efficacy of IBaCC *in vitro* against nematodes. (A) C. elegans. (Left) Photos of C. elegans N2 exposed to various conditions (spore crystal lysate [SCL] and IBaCC; both 40 µg/ml Cry5B). Scale bar, 200 µm. C. elegans was treated with azide to immobilize them just prior to imaging. (Right) Viability of C. elegans *glp-4(bn2)* L4 hermaphrodites under various conditions (IBaCC, 29 µg/ml Cry5B). *P* value for comparison is from a one-tailed *t* test. (B) Hookworm larval development. Plotted are the numbers of L3i larvae that developed from 60 hookworm eggs within 7 days (*A. ceylanicum*, left; N. americanus, right). *x* axes indicate the concentrations of Cry5B for IBaCC (IBa, 0 ng/ml for all).

IBaCC was tested and quantified for antinematode activity initially against free-living stages of nematodes. Against the free-living nematode Caenorhabditis elegans, IBaCC (containing Cry5B crystals) intoxicated and killed L4/adult stages, whereas identically prepared IBa (inactivated bacteria with empty vector control; no Cry5B) did not (Fig. 3A). When tested against the free-living developing larval stages of the human hookworms *A. ceylanicum* and N. americanus, Cry5B IBaCC, but not vector-only IBa, was highly potent, strongly inhibiting larval development even at doses of 0.5 to 1 µg/ml (Fig. 3B).

**FIG 4:**
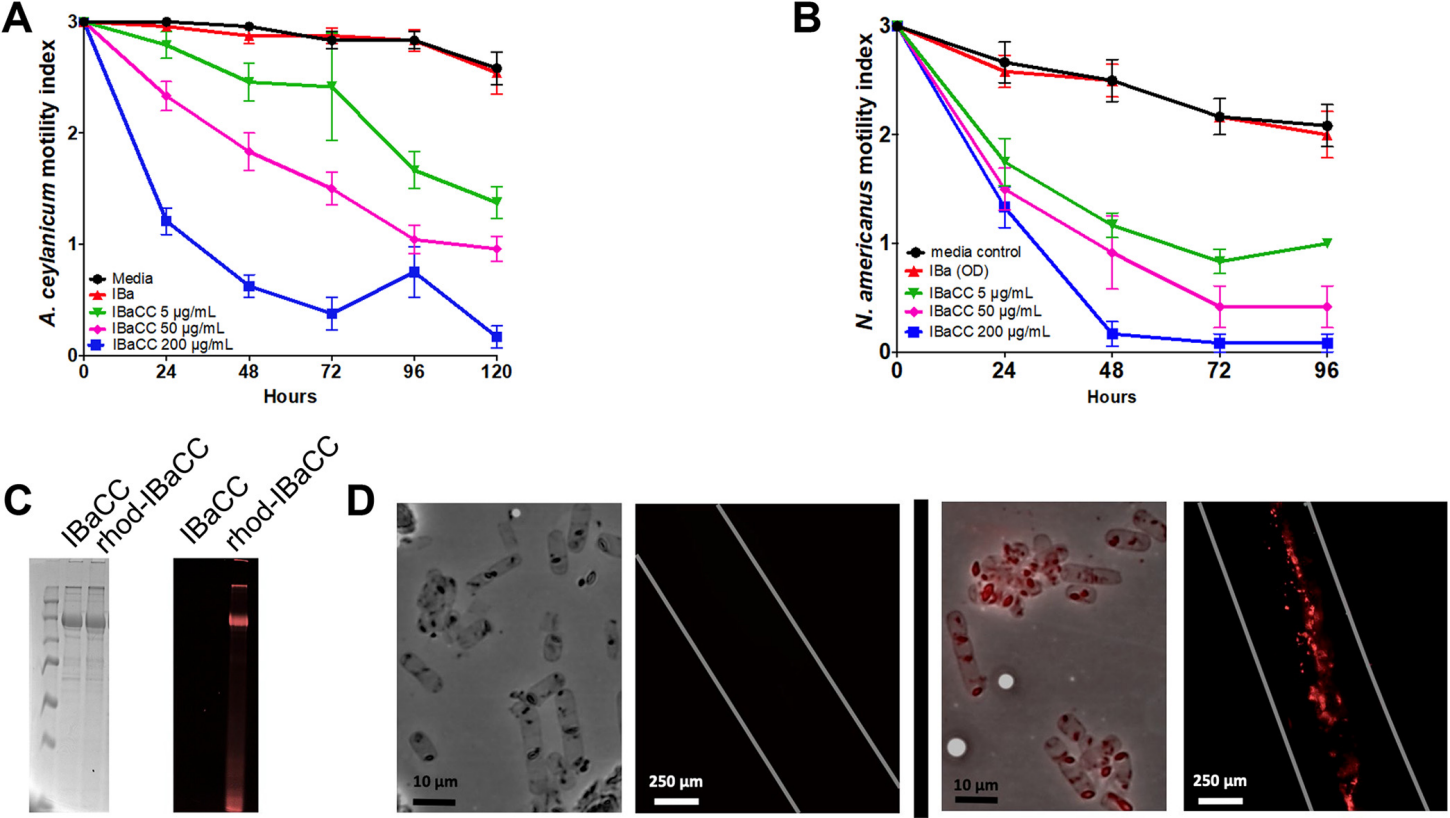
Impact of Cry5B IBaCC on adult hookworms *in vitro*. Adult hookworm motility over time at 5, 50, and 200 µg/ml Cry5B for *A. ceylanicum* adults (A) and N. americanus adults (B) *in vitro* averaged over three independent trials. In all figures, plots show averages and standard errors. (C) (Left) SDS-PAGE gel showing IBaCC before (IBaCC) and after (rhod-IBaCC) labeling with rhodamine. (Right) UV fluorescence image of same gel, showing predominant labeling of full-length Cry5B band for rhod-IBaCC. (D) Uptake of rhod-IBaCC. (Left) Bright-field image of IBaCC and rhodamine fluorescence image of adult hookworm fed IBaCC for 4 h. (Right) Bright-field image of rhod-IBaCC and rhodamine fluorescence image of adult hookworm fed rhod-IBaCC for 4 h. Uptake of rhodamine-labeled Cry5B crystals is evident.

We next tested IBaCC for antinematode activity against adult hookworm parasites *in vitro*. Cry5B IBaCC, but not IBa, was potent at intoxicating both species of adult parasitic hookworms at doses as low as 5 µg/ml (Fig. 4A and B). Cry5B in IBaCC showed a dose-dependent inhibition of motility similar to that in previously published studies with purified Cry5B (30). We had previously confirmed uptake of 0.4-µm particles by adult hookworms (50). To confirm uptake of IBaCC by hookworms, we labeled IBaCC with rhodamine, which predominantly labels full-length Cry5B (Fig. 4C). (Rhodamine-labeled IBaCC was fully potent as seen by 100% dead adult hookworms after 24 h in 16 µg/ml Cry5B rhodamine-IBaCC). Visualization of the uptake of rhodamine IBaCC after 4 h by adult hookworms *in vitro* was confirmed by fluorescence microscopy (Fig. 4D). Taken together, these data indicate that Cry5B expressed in IBaCC is ingested by, and is highly active against, nematodes, even though the bacterium is not viable. Conversely, empty vector inactivated bacteria (IBa) without Cry5B are not active against nematodes.

### Cry5B IBaCC is a potent anthelmintic *in vivo* against both genera of human hookworms.

**FIG 5:**
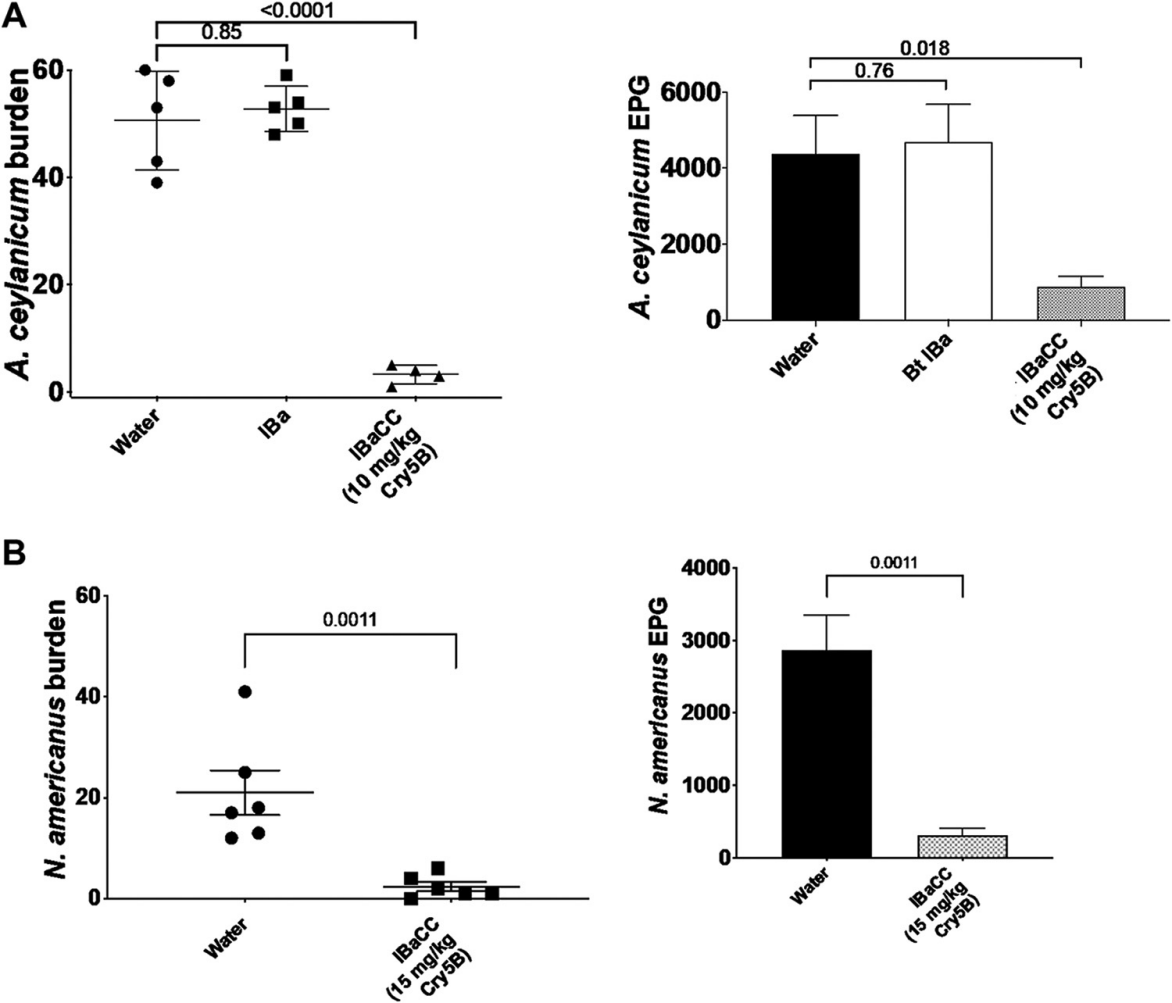
Efficacy of IBaCC produced in the laboratory *in vivo* against both human hookworm genera. (A) Shown are mean *A. ceylanicum* hookworm burdens (left) and fecal egg counts (right) in infected hamsters treated with water control, IBa, or IBaCC containing Cry5B. Here and in subsequent figures, error bars are standard errors. EPG, eggs per gram of feces. (B) Mean N. americanus hookworm burdens (left) and fecal egg counts (right) in infected hamsters treated with water control or IBaCC containing Cry5B.

We next tested whether IBaCC is efficacious *in vivo* against hookworms. Ancylostoma ceylanicum is an important zoonotic hookworm parasite of humans (51–54), and *A. ceylanicum* infections in hamsters are considered a good laboratory model for hookworm infections in humans (55). *A. ceylanicum* is also in the same genus as Ancylostoma duodenale, the second most common hookworm parasite in humans. Hamsters were infected with *A. ceylanicum*, and the infestations were allowed to proceed to mature adults with the appearance of parasite eggs (fecundity) excreted into the hamster feces (31, 33, 56). These hamsters were then treated via gavage with a single-dose of IBaCC that was produced in our laboratory containing 10 mg/kg body weight Cry5B (Fig. 5A) or treated with IBa (vector-only control produced at the same time and processed identically to Cry5B IBaCC but lacking Cry5B). The impacts of the treatment on parasite reproduction (fecal egg counts) and intestinal hookworm burdens were assessed 5 days after treatment. Whereas IBa had no impact on hookworm burdens or fecal egg counts (Fig. 5A; see also Table S2 and Fig. S1), IBaCC had a strong impact, resulting in elimination of more than 93% of the hookworms (Fig. 5A; Table S2).

We also tested Cry5B IBaCC against the most common hookworms of humans, N. americanus, which can be maintained and studied in immunosuppressed hamsters (31). This hookworm infection is more difficult to treat (e.g., *Necator*, but not *Ancylostoma*, hookworms are recalcitrant to ivermectin treatment [57]). Cry5B IBaCC was highly effective against N. americanus hookworm infections in hamsters (Fig. 5B; Table S2) (assessed 5 days after treatment).

### IBaCC is scalable and transferrable.

**FIG 6:**
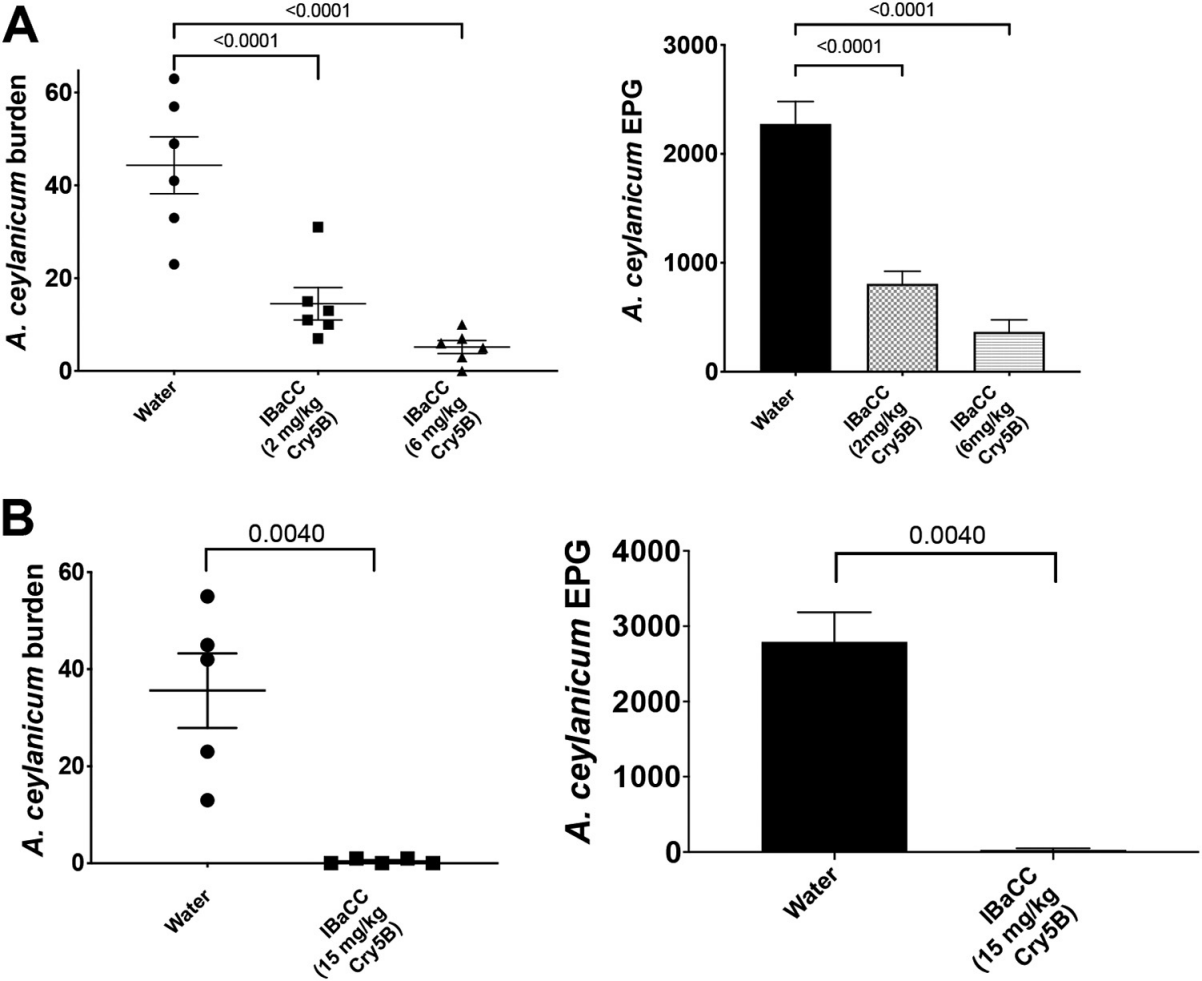
Efficacy of production facility IBaCC *in vivo* against *A. ceylanicum* hookworms. (A) Dose response of *A. ceylanicum* burdens (left) and fecal egg counts (right) in infected hamsters treated with water control or CMO-produced IBaCC containing Cry5B. (B) *A. ceylanicum* burdens (left) and fecal egg counts (right) in infected hamsters treated with water or CMO-produced 15 mg/kg Cry5B in IBaCC. EPG, eggs per gram of feces. *P* values for relevant comparisons are given.

We then looked at whether B. thuringiensis fermentation and Cry5B production and processing to IBaCC could successfully be transferred to and scaled up at a contract manufacturing facility. Our IBaCC strain and protocols were transferred to a contract manufacturing organization (CMO) at Utah State University. Fermentation of Cry5B BaCC and processing to IBaCC was brought up to the 350-liter scale by CMO. Cry5B IBaCC produced at the CMO was then tested *in vivo* against *A. ceylanicum* hookworm infestations in hamsters at 2 and 6 mg/kg body weight Cry5B. Single-dose IBaCC produced at the manufacturing facility was effective at reducing *A. ceylanicum* burdens and parasite fecal egg counts in hamsters relative to that for a water control (Fig. 6A; Table S2) (assessed 5 days after treatment). Increasing the dose of Cry5B in this large-scale IBaCC production run to 15 mg/kg essentially cured the parasite infestation (Fig. 6B; Table S2). Efficacy was similar to that for in-house produced IBaCC (Fig. 5 compared to Fig. 6).

### Cry5B IBaCC can be made into a fit-for-purpose formulation and dried down.

**FIG 7:**
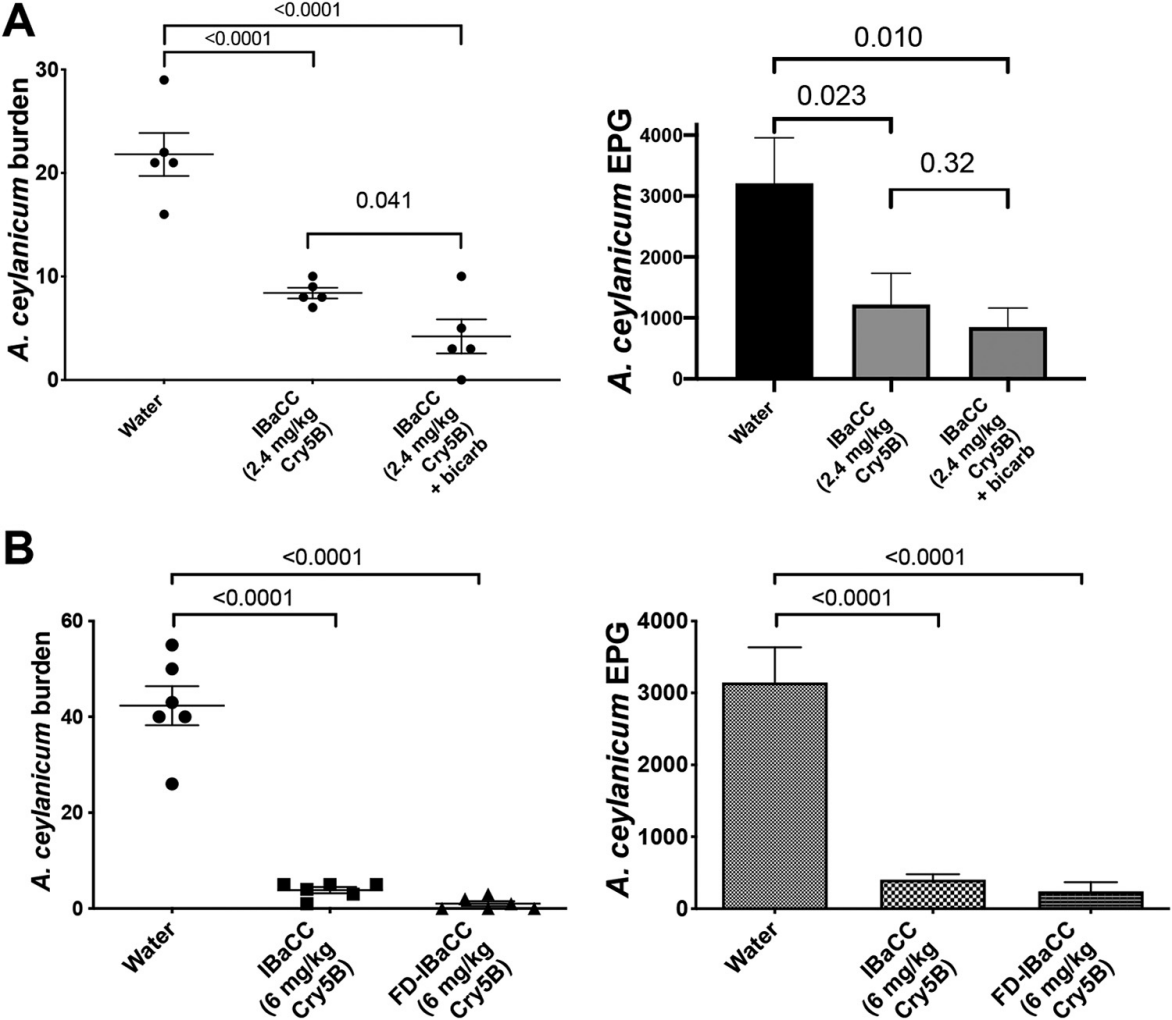
Fit-for-purpose formulation studies for Cry5B IBaCC. (A) Mean *A. ceylanicum* hookworm burdens (left) and fecal egg counts (right) in infected hamsters treated with water control, IBaCC, or IBaCC mixed with sodium bicarbonate. No cimetidine pregavage was used in these experiments. (B) Mean *A. ceylanicum* hookworm burdens (left) and fecal egg counts (right) in infected hamsters treated with water control, IBaCC, or the same batch of IBaCC freeze-dried (FD). *P* values for relevant comparisons are given.

Previous work has shown that the potency of Cry5B spore crystal lysates is enhanced by addition of a pretreatment with cimetidine to neutralize stomach acid. However, pretreatment with a drug such as cimetidine is not compatible with mass drug administration. We therefore tested whether the development of a safe and simple “fit-for-purpose” formulation compatible with mass drug administration, notably, simultaneous delivery with sodium bicarbonate, would be protective for IBaCC. IBaCC was given alone or simultaneously with sodium bicarbonate to hookworm-infected hamsters. We found that acid neutralization with sodium bicarbonate given simultaneously with IBaCC slightly but significantly increased IBaCC efficacy against hookworms (Fig. 7A; Table S2).

Delivery of IBaCC as a powder (and not as a liquid slurry as in the above-described experiments) is also a critical parameter for storage and mass drug administration (MDA). We therefore freeze-dried IBaCC into a powder and compared the efficacy of the same batch of IBaCC before and after freeze-drying (given *per os* as a powder suspension in water). As shown (Fig. 7B; Table S2), freeze-drying had no impact on IBaCC *in vivo* efficacy.

**FIG 8:**
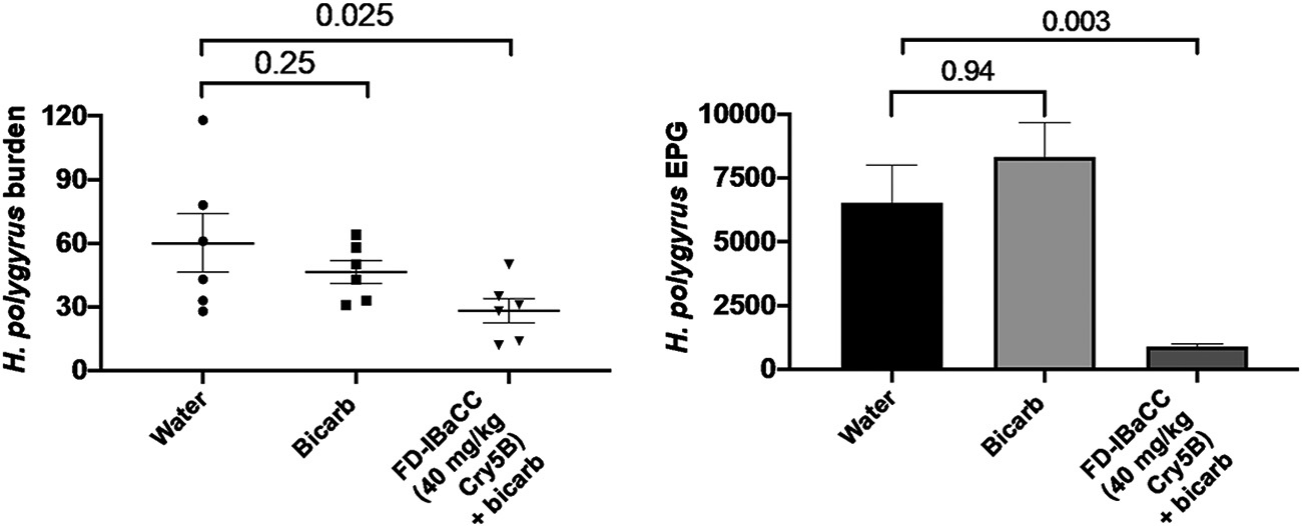
Confirmation of fit-for-purpose Cry5B IBaCC efficacy against a different, luminal feeding parasite in a second host. Mean *H. polygyrus bakeri* burdens (left) and fecal egg counts (right) in infected mice treated with water, sodium bicarbonate, or single-dose freeze-dried (FD) Cry5B IBaCC mixed with sodium bicarbonate. The FD-IBaCC used was an independent batch from that used for the data shown in Fig. 7.

Efficacy of freeze-dried IBaCC simultaneously delivered with sodium bicarbonate was also tested against a different, luminal-feeding (58) intestinal parasitic nematode in a different host, namely, Heligmosomoides polygyrus bakeri infections in mice. These data confirm that this simple fit-for-purpose IBaCC formulation is effective against a different parasitic nematode in a different rodent host (Fig. 8; Table S2). The single-dose efficacy seen (53% reduction in worm burdens and 85% reduction in fecal egg counts, single dose 40 mg/kg Cry5B) was superior to that shown in previous studies using Cry5B SCL with this parasite (31, 42) and is >20 to 80× more effective on a molar basis than ivermectin or albendazole against this same parasite (56).

### Pilot preclinical toxicology study.

Cry proteins have a stellar safety record and are considered nontoxic to vertebrates even at high doses, and the bacterium delivering IBaCC is dead (inactivated). Thus, IBaCC is predicted to be completely safe. However, IBaCC represents a new form for delivering a Cry protein. Thus, we performed a pilot maximal Cry5B dose safety study (see Fig. S2) using histopathology and blood chemistry as readouts.

Sixteen uninfected hamsters (eight female and eight male) were split into two groups. Half the hamsters (four female and four male) were given 200 mg/kg Cry5B IBaCC *per os* daily for 5 days. Since a single 40 mg/kg dose of Cry5B is curative for hookworms (31), each dose represents 5× the curative dose, and cumulatively, 5× the required number of doses were given. The other half of the hamsters received equal volumes of water *per os* on each of the 5 days. Three days after the final treatment, half of the hamsters in each group were sacrificed (acute group). All major organs were immediately dissected, fixed in formalin, sectioned, and stained, looking for signs of disease and lesions (see Materials and Methods for details). Ten days after the final treatment, the remaining half of the hamsters in each group were sacrificed (recovery group). The acute group would permit observation of any short-term adverse consequences of treatment, whereas the recovery group would permit observation of the resolution of adverse consequences seen in the acute group (if any). After staining, 274 sections from all major organs and tissues were examined in a blinded manner and scored by a board-certified pathologist.

The full results are presented in Table S3. Based on this maximal dose pilot treatment study, there were no significant differences seen between water and IBaCC groups. There were also no significant differences seen between the acute and recovery groups comparing across other groups, and no significant differences seen between males and females comparing across other groups. No significant pathologies were seen in any groups.

After sacrifice, blood samples were also collected and analyzed for blood chemistry, with a focus on enzymes that could be indicative of renal or hepatic injury (alanine aminotransferase [ALT], gamma-glutamyl transferase [GGT], aspartate aminotransferase [AST], bilirubin, blood urea nitrogen or urea, and creatinine). Comparison of hamsters in the water versus Cry5B IBaCC groups showed no statistical difference in any of these levels (see Table S4), consistent with high level of safety and lack of toxicity.

Repeated maximal dosing of IBaCC appeared to be completely safe and nontoxic to hamsters based on this pilot histopathology and blood chemistry study. Acute and chronic good laboratory practice (GLP) toxicology studies with larger sample sizes are planned in the future with clinical-grade Cry5B IBaCC.

## DISCUSSION

The primary aim of this study was to develop and demonstrate a practical scalable therapeutic for treating GIN infections in humans. The importance of these aspects of human anthelmintics are too often neglected. To date, there has been no drug developed specifically for human GINs. All drugs used for human GIN treatment came from drugs developed for veterinary targets (59). Furthermore, the availability of current drugs for human mass drug administration relies upon off-patent drugs that are donated (22). Anthelmintics for human GINs need to be effective, safe, stable, scalable, compatible with mass drug administration, and low cost.

B. thuringiensis Cry proteins, one of which has anthelmintic activity against human GINs (31, 34), have many of the characteristics required. B. thuringiensis spore crystal lysates are massively produced around the world for agriculture (75% of the biopesticide market [25]), are shelf stable, and low cost. B. thuringiensis Cry proteins have a superb track record of safety for more than 6 decades of use in agriculture (caterpillar/beetle) and vector (black fly and mosquito) control (28, 29, 60). Indeed, the specific receptor for Cry5B in nematodes is restricted to invertebrates (35). However, the use of Cry proteins as insecticidal sprays and incidental exposure in people and in >100 MHa of transgenic crops at subanthelmintic doses is a “far cry” from their use as purposely eaten therapeutics in much higher doses and as a broad-spectrum anthelmintic. For example, many commercial B. thuringiensis strains contain enterotoxin genes associated with food poisoning in B. cereus (61–63). Although strain selection could help mitigate some of this concern, there are clearly major regulatory hurdles associated with using live B. cereus family bacteria as a purposely ingested therapeutic as opposed to an agricultural spray (64, 65).

Here, for the first time, we describe a specific B. thuringiensis Cry protein form developed to deliver Cry proteins as an ingestible therapeutic to vertebrates. This new form, Cry5B IBaCC, presents the Cry5B crystal protein as a crystal contained within the cell wall “ghost” of a dead vegetative bacterium, or paraprobiotic. Cry5B IBaCC is efficacious *in vivo* against both genera of blood-feeding human hookworms and one lumen-feeding parasitic nematode, *H. polygyrus bakeri*. IBaCC and/or the crystals inside the bacterium are ingested by the parasites, causing them to be intoxicated by damage to their intestinal cells. Efficacy *in vivo* is excellent, with a near complete clearance of hookworms at 15 mg/kg. On a molar concentration scale, Cry5B IBaCC is 175× more effective at clearing hookworms than albendazole (66). High efficacy of Cry5B IBaCC against Haemonchus contortus infections in small ruminants (sheep) has also just been reported (67).

Cry5B IBaCC production was successfully transferred to an industrial CMO and scaled up to 350 liters. Cry5B IBaCC is also compatible with simple fit-for-purpose formulations, such as sodium bicarbonate (e.g., Alka-Seltzer), and can be dried down while retaining full activity. Importantly, this new form of Cry5B, IBaCC, appeared safe in a maximal multidose acute toxicology study based on histopathology and blood chemistry workups. These findings confirm the lack of Cry5B toxicity reported in all previous rodent, livestock, and companion animal studies (30–32, 34, 42). Our current focus is optimizing the Cry5B IBaCC production strain for increased Cry5B yields, scale up, and GLP toxicology studies prior to initiating first-in-human clinical trials.

By killing the Cry5B-containing vegetative bacteria, but still using the whole fermentation, a host of issues associated with live bacteria (e.g., stability, safety, and environmental contamination) or protein purification are obviated. Because the product is taken straight out of the fermenter, briefly incubated with essential oil, washed, and then ready for use, the process is simple and inexpensive. It could even be carried out locally in countries of GIN endemicity. Because the bacterium is killed, there are fewer issues with (i) degradation of the product over time as the bacterium dies, (ii) regulatory issues associated with a product that is changing over time, such as for live bacteria, as viability decays on the shelf, (iii) selection of resistance with live bacteria replicating in the environment or in the GI tract, (iv) release of live recombinant bacteria into the environment, and (v) any potential toxicity associated with live bacteria in general and with enterotoxins associated with B. cereus family of bacteria specifically (see above). These properties should make IBaCC readily acceptable to drug and environmental regulatory agencies.

These studies validate IBaCC as a powerful, practical, safe, and deployable anthelmintic compatible with MDA that can safely and effectively deliver not only Cry5B but also other antinematode B. thuringiensis Cry proteins (68) for anti-GIN therapy. IBaCC uniquely and practically harnesses the safety, massive scalability, history, and power of B. thuringiensis and B. thuringiensis Cry proteins against one of the most prevalent and intractable diseases of the poorest populations on earth. IBaCC promises new hope for a new arsenal of anthelmintics against the most common parasites of humans and animals.

## MATERIALS AND METHODS

### Nematodes.

**(i) Medium and reagents.** Reagents for hookworm culture medium (HCM), including RPMI 1640 medium, fetal bovine serum (FBS), penicillin-streptomycin, and fungizone antimycotic, were all purchased from Gibco, USA. Dexamethasone 21-phosphate disodium salt (DEX) (catalog number [cat. no.] D1159-5G) and cimetidine (cat. no. C4522-5G) were purchased from Sigma-Aldrich, USA. Cimetidine was prepared and dosed as described previously (69).

**(ii) Caenorhabditis elegans.** Caenorhabditis elegans was maintained using standard techniques (70). The following strains were used in this study: N2 Bristol (wild type) and *glp-4(bn2)*. For images shown in Fig. 3 (growth assay), hatched N2 L1 worms were incubated for 3 days at 25°C using a standard L1 growth assay (68, 71) in 48-well plates containing an Escherichia coli food source and with treatments as indicated. Assayed worms were stilled with sodium azide, washed, and arranged for imaging in spot plates. Images were taken with a dissecting microscope fitted with a camera. The bioactivity of Cry5B in freeze-dried and irradiated freeze-dried SCLs (Fig. 1) was confirmed against C. elegans by a mortality assay for 48 h at 25°C as described previously (71–73). For the Fig. 3 lethality study, assays were carried out with *glp-4(bn2)* hermaphrodites incubated at 25°C for 6 days. The use of *glp-4(bn2)*, which prevents production of progeny at the nonpermissive temperature by the hermaphrodites, is routine for these mortality assays, which would otherwise be complicated via internal hatching of larvae that sometimes occurs when adult C. elegans is intoxicated with Cry proteins (74–77). Data represent the averages and standard errors from three independent experiments with approximately 60 worms per experiment (180 total), except for the IBa control in Fig. 3A (two independent experiments).

**(iii) Animals and parasites.** Ancylostoma ceylanicum and Necator americanus life cycles were maintained as previously published (31). Three- to 4-week-old male Golden Syrian hamsters (HsdHan:AURA) were purchased from Envigo (USA) and were infected at approximately 4 to 5 weeks of age with either ∼150 *A. ceylanicum* third-stage infectious larvae (L3i) orally or ∼400 N. americanus L3i subcutaneously. Hamsters were provided with food and water *ad libitum*. The Heligmosomoides polygyrus bakeri life cycle was maintained at the United States Department of Agriculture (USDA) as described previously (78). Infectious-staged larvae were shipped to University of Massachusetts Medical School. All animal experiments were carried out under protocols approved by the University of Massachusetts Medical School. All housing and care of laboratory animals used in this study conform to the NIH Guide for the Care and Use of Laboratory Animals in Research (18-F22) and all requirements and all regulations issued by the USDA, including regulations implementing the Animal Welfare Act (P.L. 89-544) as amended (18-F23).

**(iv) *In vitro* assays with parasites.** Egg-to-larva assays were carried out as described previously (41). Adult hookworm *in vitro* assays were carried out essentially as described previously (32, 56). Briefly, for *A. ceylanicum*, three adult hookworms per well were placed in 500 µl hookworm medium in a 24-well format with the designated treatment using four wells/condition and then set up three independent times. N. americanus parasites were similarly tested but with only three wells per condition, because the number of adult parasites per hamster is more limited. For all conditions, there were roughly the same numbers of male and female worms. Hookworm adults were scored on a 0 to 3 scale (0, nonmotile even when touched; 1, nonmotile unless touched; 2, slowly motile; 3, fully motile) as described previously (32, 56).

For rhodamine-labeled IBaCC (rhod-IBaCC), IBaCC was resuspended in phosphate buffer (0.1 M, pH 7) at a concentration of 2 mg Cry5B/ml. Rhodamine isothiocyanate (RITC) was dissolved at 5 mg/ml in dimethyl sulfoxide. RITC solution (60 μl) was added to a 1-ml suspension of IBaCC, and the sample was incubated with constant mixing in the dark at room temperature for 18 h. Tris buffer (60 μl, 1 M, pH 8) was added, and the reaction mixture was stirred for an additional 15 min to quench free RITC. The sample was centrifuged to collect rhod-IBaCC, and the pellet was washed with water to remove physisorbed dye. Uptake of IBaCC and rhod-IBaCC in *A*. *ceylanicum* was evaluated at a concentration of 16 μg Cry5B/ml using three adult hookworms per well in a 24-well format. Worms were evaluated by fluorescence microscopy for particle uptake at 1.5, 4, and 24 h.

**(v) *In vivo* studies.** The *A. ceylanicum* and N. americanus *in vivo* experiments were carried out as described previously (31–33, 56). Briefly, fecal egg counts for *A. ceylanicum* were taken on days 17 to 18 postinoculation to establish groups with roughly equal infectivity, and then the animals were treated with a single-dose gavage on day 18 postinoculation. Fecal egg counts were taken again on days 22 to 23 postinoculation, and parasite burdens in the small and large intestine were assessed on day 23 postinoculation. Fecal egg counts for N. americanus were taken on days 55 to 56 postinoculation to establish groups with roughly equal infectivity, and then the animals were treated with a single-dose gavage on day 56 postinoculation. Fecal egg counts were taken again on days 60 to 61 postinoculation, and parasite burdens in the small and large intestines were assessed on day 61 postinoculation. In Fig. 5, a slightly lower dose was used against *A. ceylanicum* than against N. americanus based on previous data suggesting that the former parasite was slightly more sensitive to Cry5B treatment *in vivo* in hamsters (31). For all *in vivo* experiments, except where sodium bicarbonate was used (Fig. 7A and all of Fig. 8; see also Fig. S1 in the supplemental material), cimetidine was prepared and given *per os* 15 min ahead of Cry5B administration, as previously described (31). For experiments with sodium bicarbonate, Cry5B IBaCC was given *per os* in 200 µl 0.1 M sodium bicarbonate. Freeze-dried IBaCC was prepared as for SCL (described below). Experiments using *H. polygyrus bakeri* were carried out as described previously (31). Briefly, fecal egg counts were taken on days 14 to 15 postinoculation to establish groups with roughly equal infectivity, and then the animals were treated with a single-dose gavage on day 15 postinoculation. Fecal egg counts were taken again on days 19 to 20 postinoculation, and parasite burdens in the small and large intestines were assessed on day 20 postinoculation.

### Bacteria.

**(i) Spore and spore crystal lysates.** For the experiments shown in Fig. 1, B. thuringiensis subspecies *kurstaki* HD1-4D8 and HD1-4D9 were ordered through the *Bacillus* Genetic Stock Center. Both crystal-deficient B. thuringiensis strains were transformed with a plasmid containing the Cry5B gene (72). Spore lysates (SLs; HD1 Cry-deficient strains) and spore crystal lysates (SCLs; HD1 strains transformed with Cry5B plasmid) were prepared using standard methods (42, 72) and then stored at −80°C until use. For freeze-drying, SL and SCL samples stored at −80°C for at least 12 h were loaded into a FreeZone 1-liter benchtop freeze-dry system (Labconco catalog number 7740020). The condenser was set to −60°C and the vacuum at 22 mTor. Irradiation of freeze-dried SL and SCL was accomplished with a cobalt-60 irradiation source at the University of Massachusetts Lowell Radiation Laboratory. Irradiation doses of 5, 10, 15, 20, 25, 30, and 60 kGy were initially tested, and 15 kGy was chosen as the lowest radiation dose with a strong effect on spore viability. To determine the number of spores, 10 mg lyophilized powder was taken under sterile conditions before and after irradiation, transferred into 1 ml of sterile distilled water in microtubes, and homogenized by vortexing. One hundred microliters of the SL or SCL suspensions was removed from each sample and incubated at 80°C in a water bath for 20 min to kill any vegetative cells and then diluted by a 10-fold dilution series. One hundred microliters of diluted samples (from 10^6^ to 10^9^) was spread on top of LB agar plates in triplicates with Rattler plating beads and incubated overnight at 30°C. Colonies on each plate were manually counted.

**(ii) IBa and IBaCC strain construction and maintenance.** The promoter region of *cry3A* (48) was fused to the coding region of *cry5B* and its downstream terminator via the N-terminal ClaI restriction site. This P*_cry3A_-cry5B* expression construct was subsequently cloned into pHT3101 (79). The resulting plasmid, pHY159, was electroporated into B. thuringiensis strain 407 Δ*spo0A*::*kan* (49). The entire *cry5B* insert was sequenced to confirm no mutations were included. Single colonies of 407 Δ*spo0A*::*kan* cells harboring pHY159 or pHT3101 empty vector control (EVC) were grown in nutrient-rich 3× LB with erythromycin (10 μg/ml) at 30°C with shaking at 250 rpm for 2 to 3 days. Outsourced cultures at a biomanufacturing facility were similarly grown in a fermenter with constant monitoring and adjustments of pH and oxygen levels at 30°C with 150 rpm agitation for 48 h. In both cases, cells were harvested by centrifugation and resuspended to 10% initial volumes in ice-cold water. Tenfold concentrations of harvested cultures were inactivated with the addition of food-grade monoterpene or essential oil at 0.1% final concentration and incubated with gentle agitation at room temperature for 15 min. Inactivated cultures were then centrifuged, washed with water, and resuspended in saline at 10% the initial volume. For all experiments except those shown in Fig. 7B, residual terpenes were extracted with corn oil (20% final volume) with gentle agitation at room temperature for 2 h. We have not found any impact on *in vivo* efficacy with or without this step. IBaCC was recovered after centrifugation and three washes with ice-cold water. Concentrated (10×) samples were removed for several analyses, including SDS-PAGE (for Cry5B content and quantification), cell density, cell viability, and nematode-killing assays.

For experiments shown in Table S1, B. thuringiensis 407 Δ*spo0A*::*kan* cells transferred with pHY159 were grown in Luria broth overnight at 30°C in 10 µg/ml erythromycin to saturation. The next day, 100 µl of terpene or essential oil (80) was added to 100 µl of overnight culture to a final volume of 1 ml in Luria broth plus erythromycin (the following terpenes and essential oil used were: geraniol, eugenol, thymol, citral, carvacrol, cinnamic aldehyde, tea tree, limonene, and undecanoic acid). Tubes were shaken overnight at 30°C. The next day, the cells were pelleted by centrifugation, washed with sterile water three times, and resuspended in 1 ml of sterile water. From each sample, 25 µl was plated onto a Luria broth plate and incubated at 30°C overnight. Growth or lack of growth was noted the following day.

### Histopathology and blood chemistry.

Organs from euthanized male and female animals were fixed in 10% neutral buffered formalin, processed, embedded in paraffin, sectioned at 5 μm, and stained with hematoxylin and eosin at Tufts University, Cummings School of Veterinary Medicine, Core Histology Laboratory (North Grafton, MA, USA). Hematoxylin stains nuclei and structures rich in nucleic acids gray to blue to dark purple. Eosin stains protein-rich regions of cells and tissues various shades of pink. All sections were examined in a blinded manner by a board-certified veterinary pathologist (G.B.). The organs sampled (and number sections examined) per animal included the following: stomach (2), small intestine (4), pancreas (1), large intestine (2 to 3), kidney (2), adrenal (1), liver (3), spleen (1), mesentery (1), brain (4 to 6), lungs (3 to 4), heart (entire), thymus (1 to 2), cortical bone (multiple), bone marrow (multiple), and growth plate (multiple). All tissues were examined for the following signs of microscopic disease and lesions: presence of nematodes/eggs, cellular immune or inflammatory infiltrates, cellular degeneration, apoptosis, and necrosis, lesion severity (none, minimal, moderate, or severe), lesion location (anatomic site), and incidental findings. After examination without knowledge of the groups, the study key was provided. In all animals and in all groups (males, females, IBaCC, and water), the following tissues were considered within normal limits by light microscopy: lymphoid or hematopoietic tissues (thymus, spleen, Peyer’s patches, and bone marrow), central nervous system (brain), endocrine (adrenal), skeletal (bone and growth plates), cardiopulmonary (heart and lungs), upper digestive tract (squamous portion of the stomach, liver, or pancreas), and lower digestive tract (large intestine). The small intestines of all animals in all treatment groups contained minimal-to-mild, multifocal, plasmocytic to lymphoplasmocytic, and rarely eosinophilic infiltrates within the lamina propria. The infiltrates were interpreted as normal resident mucosal immune cells. There was no evidence of significant inflammation, degeneration, necrosis, fibrosis, or other toxicities in these tissues. Multifocal, minimal-to-mild mineralized foci were noted in the glandular epithelium of the stomach and the kidneys (renal tubules and collecting ducts) of all males and females in both the IBaCC and water groups, indicating it is not a consequence of treatment. These mineralized foci are known background lesions of laboratory hamsters (81). The glandular stomachs of females and males in both treatment groups contained particulates in the superficial mucus, of uncertain cause or significance. In a fraction of animals in all treatment groups (1 of 4 IBaCC-treated females; 2 of 3 water-treated females; 3 of 3 IBaCC males, and 2 of 4 water-treated males), minimal-to-mild lymphocytic serositis with reactive mesothelial cell hypertrophy was observed. The cause and clinical significance of this lesion was not apparent, but the lesion does not appear to be a specific adverse effect attributable to IBaCC treatment. None of these hamsters were infected and, indeed, in no section were any nematodes or nematode eggs seen.

Immediately after euthanasia, hamsters were exsanguinated by cardiac puncture, and blood was collected into SAFE-T-FILL capillary blood collection tubes-serum (RAM Scientific). Blood was allowed to clot for at least 30 min at room temperature before centrifugation. The collected serum was stored at −80°C until further use. Serum biochemistry profiles were performed on a COBAS c501 chemistry analyzer (Roche Diagnostics, Indianapolis, IN, USA) using standard protocols.

### Statistical analyses.

Prism v. 7 was used for all graphs and two-group comparisons. Multigroup comparisons were carried out with SPSS v. 25. For all comparisons, including just two groups, except for the C. elegans data (due to only three samples per group), a one-tailed Mann-Whitney (nonparametric) test was used with the assumption that treatment reduced parasite burdens and fecal egg counts. For the C. elegans data, a one-tailed Student’s *t* test was used. For all comparisons involving more than one group, one-way analysis of variance (ANOVA) with a one-tailed Dunnett’s posttest was used to compare groups relative to control or with Sidak’s posttest for comparison between other groups.

## Supplementary Material

Supplemental file 1
