# Supplementary material for: Recombinant Paraprobiotics as a New Paradigm for Treating Gastrointestinal Nematode Parasites of Humans
Source: Antimicrob Agents Chemother. 2021 Feb 17;65(3):e01469-20. doi: 10.1128/AAC.01469-20 (PMC8092541; doi:10.1128/AAC.01469-20)
Supplement: Supplemental file 1 [file AAC.01469-20-s0001.pdf]

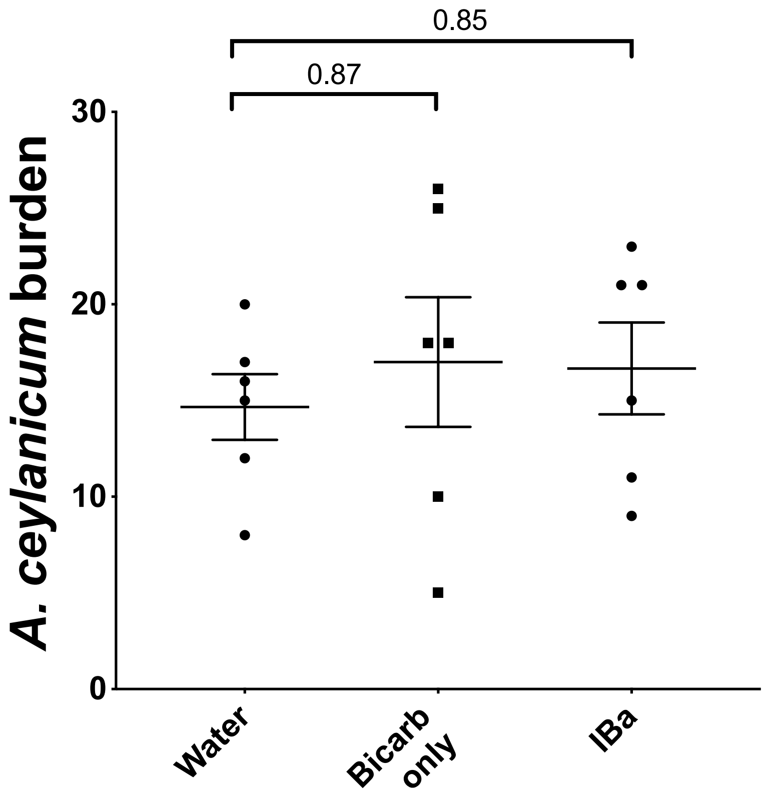

Figure S1. Negative control studies for hookworm burdens. Neither sodium bicarbonate alone nor IBa significantly impact hookworm burdens in hamsters.

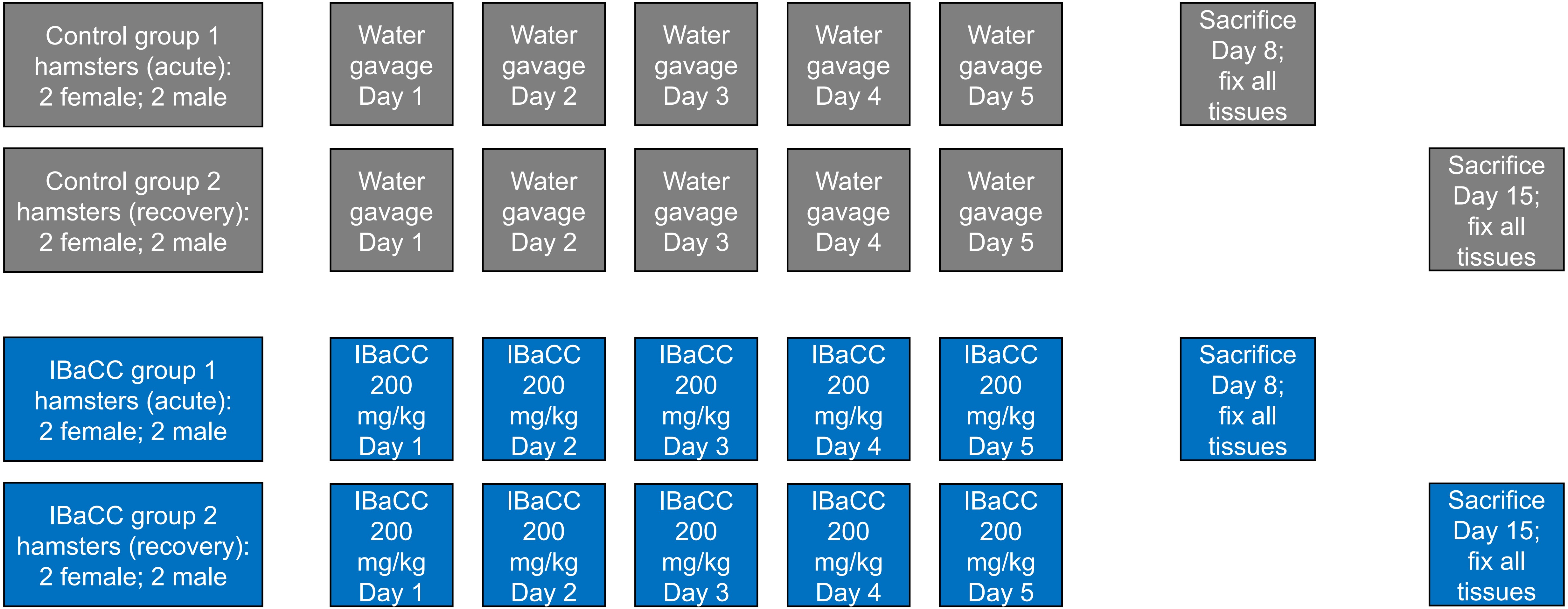

Figure S2. Experimental design for preliminary safety study. Volume of gavage was the same in all groups.

Table S1. Qualitative survey of essential oils tested against BaCC cells.

Bt Spo0A- cells present?	Terpene tested (1 mg/ml)	Bt SpoA- dilution	Growth on plates?
NO	none	NA	NO
YES	none	1.00E-03	YES
YES	Geraniol	1.00E-03	NO
YES	Eugenol	1.00E-03	NO
YES	Thymol	1.00E-03	NO
YES	Citral	1.00E-03	NO
YES	Carvacrol	1.00E-03	NO
YES	Cinnamic ald	1.00E-03	NO
YES	Tea tree	1.00E-03	YES
YES	Limonene	1.00E-03	YES
YES	Undecanoic acid	1.00E-03	NO

Table S2. Numerical values of worm burdens and fecal egg counts for all *in vivo* studies.

Figure	Condition	Average worm burden (n)	Change from control	Average EPG (n)	Change from control
5A	Water	50.6 (5)	NA	4390 (5)	NA
	IBa	52.8 (5)	4.30%	4695 (5)	6.90%
	IBaCC 10 mg/kg Cry5B	3.3 (4)	-93.50%	875.0 (4)	-80.10%
5B	Water	21.0 (6)	NA	2867 (6)	NA
	IBaCC 15 mg/kg Cry5B	2.3 (6)	-89.00%	308.3 (6)	-89.20%
6A	Water	44.3 (6)	NA	2275 (6)	NA
	IBaCC 2 mg/kg Cry5B	14.5 (6)	-67.30%	808.3 (6)	-64.50%
	IBaCC 6 mg/kg Cry5B	5.2 (6)	-88.30%	366.7 (6)	-83.80%
6B	Water	35.6 (5)	NA	2790 (5)	NA
	IBaCC 15 mg/kg Cry5B	0.4 (5)	-98.90%	30.0 (5)	-98.90%
7A	Water	21.8 (5)	NA	3210 (5)	NA
	IBaCC 2.4 mg/kg Cry5B	8.4 (5)	-61.50%	1220 (5)	-62.00%
	IBaCC 2.4 mg/kg Cry5B + Bicarb	4.2 (5)	-80.70%	850.0 (5)	-73.50%
7B	Water	42.3 (6)	NA	3146 (6)	NA
	IBaCC 6 mg/kg Cry5B	3.8 (6)	-91.00%	404.2 (6)	-87.20%
	FD-IBaCC 6 mg/kg Cry5B	1.0 (6)	-97.60%	241.7 (6)	-92.30%
8	Water	60.2 (6)	NA	6517 (6)	NA
	Bicarb	46.5 (6)	-22.80%	8325 (6)	27.70%
	FD-IBaCC 40 mg/kg Cry5B + Bicarb	28.3 (6)	-53.00%	883.3 (6)	-86.40%
Supplement S1.	Water	14.7 (6)	NA		
	Bicarb only	17.0 (6)	15.60%		
	IBa	16.7 (6)	13.60%		

Table S3. Histopathology results. na=not applicable.

Study name	Animal type	Age (weeks)	Gender	Treatment type	Route of treatment	Day of euthanasia (after last dose)	slide label	organ	diagnosis	lesion	lesion type	severity	distribution	location(s)	cell types	incidental finding	Comments
IBaCCHistoPath1	Hamster	8	F	IBACC	gavage	10	A5.1	stomach, squamous	na	na	na	na	na	na	na	na	na
IBaCCHistoPath1	Hamster	8	F	IBACC	gavage	10	A5.1	stomach, glandular	ingested material in mucus	y	incidental	na	diffuse	mucus	na	na	na
IBaCCHistoPath1	Hamster	8	F	IBACC	gavage	10	A5.1	SI	eosinophilic and lymphoplasmacytic infiltrates	n	na	minimal	mf	lamina propria	eosinophils, lymphocytes, plasma calle	n	expected variation in resident immune cells
IBaCCHistoPath1	Hamster	8	M	IBACC	gavage	10	A1 .1	Peyer's patches	normal	n	na	na	na	na	na	na	na
IBaCCHistoPath1	Hamster	8	M	IBACC	gavage	10	A1 .1	pancreas	normal	n	na	na	na	na	na	na	na
IBaCCHistoPath1	Hamster	8	M	IBACC	gavage	10	A1 .1	LI	normal	n	na	na	na	na	na	na	na
IBaCCHistoPath1	Hamster	8	F	IBACC	gavage	10	A6.1	stomach, squamous	na	na	na	na	na	na	na	na	na
IBaCCHistoPath1	Hamster	8	F	IBACC	gavage	10	A6.1	stomach, glandular	mucosal mineralized foci	y	foreign material in mucus; mineralized foci;	mild	mf	mucus; mucosa	na	na	na
IBaCCHistoPath1	Hamster	8	F	IBACC	gavage	10	A6.1	SI	plasma cell infiltrates	n	na	minimal	mf	lamina propria	plasma cells	n	expected variation in resident immune cells
IBaCCHistoPath1	Hamster	8	M	IBACC	gavage	10	A2.1	Peyer's patches	na	na	na	na	na	na	na	na	na
IBaCCHistoPath1	Hamster	8	M	IBACC	gavage	10	A2.1	pancreas	normal	n	na	na	na	na	na	na	na
IBaCCHistoPath1	Hamster	8	M	IBACC	gavage	10	A2.1	LI	normal	n	na	na	na	na	na	na	na
IBaCCHistoPath1	Hamster	8	F	WATER	gavage	10	A8.1	stomach, squamous	na	na	na	na	na	na	na	na	na
IBaCCHistoPath1	Hamster	8	F	WATER	gavage	10	A7.1	stomach, glandular	mucosal mineralized foci	y	mineralized foci	mild	mf	mucosa	na	y	na
IBaCCHistoPath1	Hamster	8	F	WATER	gavage	10	A7.1	SI	eosinophilic and plasmacytic infiltrates	n	na	minimal	mf	lamina propria	eosinophils & plasma cells	n	expected variation in resident immune cells
IBaCCHistoPath1	Hamster	8	M	WATER	gavage	10	A3.1	Peyer's patches	normal	n	na	na	na	na	na	na	na
IBaCCHistoPath1	Hamster	8	M	WATER	gavage	10	A3.1	pancreas	normal	n	na	na	na	na	na	na	na
IBaCCHistoPath1	Hamster	8	M	WATER	gavage	10	A3.1	LI	normal	n	na	na	na	na	na	na	na
IBaCCHistoPath1	Hamster	8	F	IBACC	gavage	3	B1.1	stomach, squamous	na	na	na	na	na	na	na	na	na
IBaCCHistoPath1	Hamster	8	F	WATER	gavage	10	A8.1	stomach, glandular	ingested material in mucus; mucosal mineralized foci	y	mineralized foci	minimal	mf	mucus; mucosa	na	y	na
IBaCCHistoPath1	Hamster	8	F	WATER	gavage	10	A8.1	SI	eosinophilic and plasmacytic infiltrates	n	na	minimal	mf	lamina propria	eosinophils & plasma cells	n	expected variation in resident immune cells
IBaCCHistoPath1	Hamster	8	M	WATER	gavage	10	A4.1	Peyer's patches	normal	n	na	na	na	na	na	na	na
IBaCCHistoPath1	Hamster	8	M	WATER	gavage	10	A4.1	pancreas	normal	n	na	na	na	na	na	na	na
IBaCCHistoPath1	Hamster	8	M	WATER	gavage	10	A4.1	LI	normal	n	na	na	na	na	na	na	na
IBaCCHistoPath1	Hamster	8	F	WATER	gavage	3	B4.1	stomach, squamous	na	na	na	na	na	na	na	na	na
IBaCCHistoPath1	Hamster	8	F	IBACC	gavage	3	B1.1	stomach, glandular	ingested material in mucus; mucosal mineralized foci	y	mineralized foci	mild	mf	na	na	y	na
IBaCCHistoPath1	Hamster	8	F	IBACC	gavage	3	B1.1	SI	plasma cell infiltrates	n	na	minimal	mf	lamina propria	plasma cells	n	expected variation in resident immune cells
IBaCCHistoPath1	Hamster	8	F	IBACC	gavage	10	A5.1	Peyer's patches	normal	n	na	na	na	na	na	na	na
IBaCCHistoPath1	Hamster	8	F	IBACC	gavage	10	A5.1	pancreas	normal	n	na	na	na	na	na	na	na
IBaCCHistoPath1	Hamster	8	F	IBACC	gavage	10	A5.1	LI	normal	n	na	na	na	na	na	na	na
IBaCCHistoPath1	Hamster	8	F	WATER	gavage	10	A7.1	stomach, squamous	normal	n	na	na	na	na	na	na	na
IBaCCHistoPath1	Hamster	8	F	IBACC	gavage	3	B2.1	stomach, glandular	ingested material in mucus; mucosal mineralized foci	y	mineralized foci	mild	mf	mucus; mucosa	na	y	na
IBaCCHistoPath1	Hamster	8	F	IBACC	gavage	3	B2.1	SI	plasma cell infiltrates	n	na	minimal	mf	lamina propria	plasma cells	n	expected variation in resident immune cells
IBaCCHistoPath1	Hamster	8	F	IBACC	gavage	10	A6.1	Peyer's patches	na	na	na	na	na	na	na	na	na
IBaCCHistoPath1	Hamster	8	F	IBACC	gavage	10	A6.1	pancreas	na	na	na	na	na	na	na	na	na
IBaCCHistoPath1	Hamster	8	F	IBACC	gavage	10	A6.1	LI	normal	n	na	na	na	na	na	na	na
IBaCCHistoPath1	Hamster	8	F	IBACC	gavage	3	B2.1	stomach, squamous	normal	n	na	na	na	na	na	na	na
IBaCCHistoPath1	Hamster	8	F	WATER	gavage	3	B3.1	stomach, glandular	mucosal mineralized foci	y	mineralized foci	mild	mf	mucosa	na	y	na
IBaCCHistoPath1	Hamster	8	F	WATER	gavage	3	B3.1	SI	eosinophilic and lymphoplasmacytic infiltrates	n	na	mild	mf	lamina propria	plasma cells	n	expected variation in resident immune cells
IBaCCHistoPath1	Hamster	8	F	WATER	gavage	10	A7.1	Peyer's patches	normal	n	na	na	na	na	na	na	na
IBaCCHistoPath1	Hamster	8	F	WATER	gavage	10	A7.1	pancreas	normal	n	na	na	na	na	na	na	na
IBaCCHistoPath1	Hamster	8	F	WATER	gavage	10	A7.1	LI	normal	n	na	na	na	na	na	na	na
IBaCCHistoPath1	Hamster	8	F	WATER	gavage	3	B3.1	stomach, squamous	normal	n	na	na	na	na	na	na	na
IBaCCHistoPath1	Hamster	8	F	WATER	gavage	3	B4.1	stomach, glandular	mucosal mineralized foci; multifocal mineralization of small arterioles	y	mineralized foci	mild; minimal	mf	mucosa; small arterioles in lamina propria/muscularis mucosa	endothelial cells & smooth muscle	y	na
IBaCCHistoPath1	Hamster	8	F	WATER	gavage	3	B4.1	SI	plasma cell infiltrates	n	na	minimal	mf	lamina propria	plasma cells	n	expected variation in resident immune cells
IBaCCHistoPath1	Hamster	8	F	WATER	gavage	10	A8.1	Peyer's patches	normal	n	na	na	na	na	na	na	na
IBaCCHistoPath1	Hamster	8	F	WATER	gavage	10	A8.1	pancreas	normal	n	na	na	na	na	na	na	na
IBaCCHistoPath1	Hamster	8	F	WATER	gavage	10	A8.1	LI	normal	n	na	na	na	na	na	na	na
IBaCCHistoPath1	Hamster	8	F	IBACC	gavage	3	B2.1	adrenal	normal	n	na	na	na	na	na	na	na
IBaCCHistoPath1	Hamster	8	F	IBACC	gavage	10	A5.3	kidney	mineralized foci	y	mineralized foci	mild	mf	collecting ducts	epithelial cell	y	na
IBaCCHistoPath1	Hamster	8	M	IBACC	gavage	10	A2.1	spleen	normal	n	na	na	na	na	na	na	na
IBaCCHistoPath1	Hamster	8	F	IBACC	gavage	10	A5.5	mesentery	lymphocytic serositis with mesothelial hypertrophy	y	inflammation, hypertrophy	minimal	focal	mesentery	lymphocytes, mesothelial cells	incidental	incidental
IBaCCHistoPath1	Hamster	8	F	WATER	gavage	3	B3.2	adrenal	normal	n	na	na	na	na	na	na	na
IBaCCHistoPath1	Hamster	8	F	IBACC	gavage	10	A6.2	kidney	mineralized foci	y	mineralized foci	minimal	focal	collecting ducts	epithelial cell	y	na
IBaCCHistoPath1	Hamster	8	M	IBACC	gavage	10	A2.2	spleen	normal	n	na	na	na	na	na	na	na
IBaCCHistoPath1	Hamster	8	F	IBACC	gavage	10	A6.2	mesentery	normal	n	na	na	na	na	na	na	na
IBaCCHistoPath1	Hamster	8	M	IBACC	gavage	10	A1.5	bone marrow	normal	n	na	na	na	na	na	na	na
IBaCCHistoPath1	Hamster	8	F	WATER	gavage	10	A7.2	kidney	mineralized foci	y	mineralized foci	minimal	mf	collecting ducts	epithelial cell	y	na
IBaCCHistoPath1	Hamster	8	M	WATER	gavage	10	A3.2	spleen	normal	n	na	na	na	na	na	na	na
IBaCCHistoPath1	Hamster	8	F	WATER	gavage	10	A7.2	mesentery	na	na	na	na	na	na	na	na	na
IBaCCHistoPath1	Hamster	8	M	IBACC	gavage	10	A2.5	bone marrow	normal	n	na	na	na	na	na	na	na
IBaCCHistoPath1	Hamster	8	F	WATER	gavage	10	A8.2	kidney	mineralized foci	y	mineralized foci	minimal	mf	collecting ducts	epithelial cell	y	na
IBaCCHistoPath1	Hamster	8	M	WATER	gavage	10	A4.2	spleen	normal	n	na	na	na	na	na	na	na
IBaCCHistoPath1	Hamster	8	F	WATER	gavage	10	A8.2	mesentery	lymphophohistiocytic serositis with mesothelial hypertrophy	y	inflammation, hypertrophy	moderate	mf	mesentery	lymphocytes, mesothelial cells	incidental	incidental
IBaCCHistoPath1	Hamster	8	M	WATER	gavage	10	A3.5	bone marrow	normal	n	na	na	na	na	na	na	na
IBaCCHistoPath1	Hamster	8	F	IBACC	gavage	3	B2.1	kidney	mineralized foci	y	mineralized foci	minimal	mf	glomeruli/Bowman's capcule	endothelial	y	na
IBaCCHistoPath1	Hamster	8	F	IBACC	gavage	10	A5.4	spleen	normal	n	na	na	na	na	na	na	na
IBaCCHistoPath1	Hamster	8	F	IBACC	gavage	3	B2.1	mesentery	normal	n	na	na	na	na	na	na	na
IBaCCHistoPath1	Hamster	8	M	WATER	gavage	10	A4.5	bone marrow	normal	n	na	na	na	na	na	na	na
IBaCCHistoPath1	Hamster	8	F	IBACC	gavage	3	B2.2	kidney	equivocal mineralization of basement membranes	y	mineralized foci	equivoval	mf	Bowman's capsule	na	y	na
IBaCCHistoPath1	Hamster	8	F	IBACC	gavage	10	A6.2	spleen	normal	n	na	na	na	na	na	na	na
IBaCCHistoPath1	Hamster	8	F	IBACC	gavage	3	B2.2	mesentery	normal	n	na	na	na	na	na	na	na
IBaCCHistoPath1	Hamster	8	F	IBACC	gavage	10	A5.5	bone marrow	normal	n	na	na	na	na	na	na	na
IBaCCHistoPath1	Hamster	8	F	WATER	gavage	3	B3.2	kidney	singe mineralized focus	y	mineralized foci	minimal	focal	collecting ducts	epithelial cell	y	na
IBaCCHistoPath1	Hamster	8	F	WATER	gavage	10	A7.2	spleen	normal	n	na	na	na	na	na	na	na
IBaCCHistoPath1	Hamster	8	F	WATER	gavage	3	B3.2	mesentery	lymphocytic serositis	y	inflammation	minimal	focal	mesentery	lymphocytes	incidental	incidental
IBaCCHistoPath1	Hamster	8	F	IBACC	gavage	10	A6.5	bone marrow	normal	n	na	na	na	na	na	na	na
IBaCCHistoPath1	Hamster	8	F	WATER	gavage	3	B4.2	kidney	mineralized foci	y	mineralized foci	mild	mf	collecting ducts	epithelial cell	y	na
IBaCCHistoPath1	Hamster	8	F	WATER	gavage	10	A8.2	spleen	normal	n	na	na	na	na	na	na	na
IBaCCHistoPath1	Hamster	8	F	WATER	gavage	3	B4.2	mesentery	normal	n	na	na	na	na	na	na	na
IBaCCHistoPath1	Hamster	8	F	WATER	gavage	10	A7.5	bone marrow	normal	n	na	na	na	na	na	na	na
IBaCCHistoPath1	Hamster	8	F	WATER	gavage	10	A8.5	bone marrow	normal	n	na	na	na	na	na	na	na
IBaCCHistoPath1	Hamster	8	F	IBACC	gavage	3	B1.5	bone marrow	normal	n	na	na	na	na	na	na	na
IBaCCHistoPath1	Hamster	8	F	IBACC	gavage	3	B2.5	bone marrow	normal	n	na	na	na	na	na	na	na
IBaCCHistoPath1	Hamster	8	F	WATER	gavage	3	B3.5	bone marrow	normal	n	na	na	na	na	na	na	na
IBaCCHistoPath1	Hamster	8	F	WATER	gavage	3	B4.5	bone marrow	normal	n	na	na	na	na	na	na	na
IBaCCHistoPath1	Hamster	8	M	IBACC	gavage	3	B5.5	bone marrow	normal	n	na	na	na	na	na	na	na
IBaCCHistoPath1	Hamster	8	M	IBACC	gavage	3	B6.5	bone marrow	normal	n	na	na	na	na	na	na	na
IBaCCHistoPath1	Hamster	8	M	IBACC	gavage	10	A1.4	lungs	congestion/hemorrhage	y	hemorrhage	moderate	mf	lung	red blood cells	agonal change	na
IBaCCHistoPath1	Hamster	8	M	WATER	gavage	3	B7.5	bone marrow	normal	n	na	na	na	na	na	na	na
IBaCCHistoPath1	Hamster	8	F	IBACC	gavage	10	A5.4	thymus	normal	n	na	na	na	na	na	na	na
IBaCCHistoPath1	Hamster	8	M	IBACC	gavage	10	A2.4	lungs	normal	n	na	na	na	na	na	na	na
IBaCCHistoPath1	Hamster	8	M	WATER	gavage	3	B7.8	bone marrow	normal	n	na	na	na	na	na	na	na
IBaCCHistoPath1	Hamster	8	F	IBACC	gavage	10	A6.4	thymus	normal	n	na	na	na	na	na	na	na
IBaCCHistoPath1	Hamster	8	M	WATER	gavage	10	A3.4	lungs	normal	n	na	na	na	na	na	na	na
IBaCCHistoPath1	Hamster	8	M	IBACC	gavage	10	A1.3	brain	normal	n	na	na	na	na	na	glial rests	na
IBaCCHistoPath1	Hamster	8	F	WATER	gavage	10	A7.4	thymus	normal	n	na	na	na	na	na	na	na
IBaCCHistoPath1	Hamster	8	M	WATER	gavage	10	A4.6	lungs	normal	n	na	na	na	na	na	na	na
IBaCCHistoPath1	Hamster	8	M	IBACC	gavage	10	A2.3	brain	normal	n	na	na	na	na	na	glial rests	na
IBaCCHistoPath1	Hamster	8	F	WATER	gavage	10	A8.4	thymus	normal	n	na	na	na	na	na	na	na
IBaCCHistoPath1	Hamster	8	F	IBACC	gavage	10	A5.4	lungs	atelectasis	n	na	na	na	na	na	na	na
IBaCCHistoPath1	Hamster	8	M	WATER	gavage	10	A3.3	brain	normal	n	na	na	na	na	na	glial rests	na
IBaCCHistoPath1	Hamster	8	F	IBACC	gavage	3	B1.4	thymus	normal	n	na	na	na	na	na	na	na
IBaCCHistoPath1	Hamster	8	F	IBACC	gavage	10	A6.4	lungs	normal	n	na	na	na	na	na	na	na
IBaCCHistoPath1	Hamster	8	M	WATER	gavage	10	A4.3	brain	normal	n	na	na	na	na	na	glial rests	na
IBaCCHistoPath1	Hamster	8	F	IBACC	gavage	3	B2.4	thymus	normal	n	na	na	na	na	na	na	na
IBaCCHistoPath1	Hamster	8	F	WATER	gavage	10	A7.4	lungs	atelectasis	n	na	na	na	na	na	na	na
IBaCCHistoPath1	Hamster	8	F	IBACC	gavage	10	A5.3	brain	normal	n	na	na	na	na	na	glial rests	na
IBaCCHistoPath1	Hamster	8	F	WATER	gavage	3	B3.4	thymus	na	na	na	na	na	na	na	na	na
IBaCCHistoPath1	Hamster	8	F	WATER	gavage	10	A8.4	lungs	atelectasis	n	na	na	na	na	na	na	na
IBaCCHistoPath1	Hamster	8	F	IBACC	gavage	10	A6.3	brain	normal	n	na	na	na	na	na	glial rests	na
IBaCCHistoPath1	Hamster	8	F	WATER	gavage	3	B4.4	thymus	normal	n	na	na	na	na	na	na	na
IBaCCHistoPath1	Hamster	8	F	WATER	gavage	10	A7.3	brain	normal	n	na	na	na	na	na	glial rests	na
IBaCCHistoPath1	Hamster	8	F	WATER	gavage	10	A8.3	brain	normal	n	na	na	na	na	na	glial rests	na
IBaCCHistoPath1	Hamster	8	F	IBACC	gavage	3	B1.3	brain	normal	n	na	na	na	na	na	glial rests	na
IBaCCHistoPath1	Hamster	8	F	IBACC	gavage	3	B2.3	brain	normal	n	na	na	na	na	na	glial rests	na
IBaCCHistoPath1	Hamster	8	F	WATER	gavage	3	B3.3	brain	normal	n	na	na	na	na	na	glial rests	na
IBaCCHistoPath1	Hamster	8	F	WATER	gavage	3	B4.3	brain	normal	n	na	na	na	na	na	glial rests	na
IBaCCHistoPath1	Hamster	8	M	IBACC	gavage	3	B5.3	brain	normal	n	na	na	na	na	na	glial rests	na
IBaCCHistoPath1	Hamster	8	M	IBACC	gavage	3	B6.3	brain	normal	n	na	na	na	na	na	glial rests	na
IBaCCHistoPath1	Hamster	8	M	WATER	gavage	3	B7.3	brain	normal	n	na	na	na	na	na	glial rests	na
IBaCCHistoPath1	Hamster	8	M	WATER	gavage	3	B8.3	brain	normal	n	na	na	na	na	na	glial rests	na
IBaCCHistoPath1	Hamster	8	M	IBACC	gavage	10	A1.5	cortical bone	normal	n	na	na	na	na	na	na	na
IBaCCHistoPath1	Hamster	8	M	IBACC	gavage	10	A2.5	cortical bone	normal	n	na	na	na	na	na	na	na
IBaCCHistoPath1	Hamster	8	M	WATER	gavage	10	A3.5	cortical bone	normal	n	na	na	na	na	na	na	na
IBaCCHistoPath1	Hamster	8	M	WATER	gavage	10	A4.5	cortical bone	normal	n	na	na	na	na	na	na	na
IBaCCHistoPath1	Hamster	8	F	IBACC	gavage	10	A5.5	cortical bone	normal	n	na	na	na	na	na	na	na
IBaCCHistoPath1	Hamster	8	F	IBACC	gavage	10	A6.5	cortical bone	normal	n	na	na	na	na	na	na	na
IBaCCHistoPath1	Hamster	8	F	WATER	gavage	10	A7.5	cortical bone	normal	n	na	na	na	na	na	na	na
IBaCCHistoPath1	Hamster	8	F	WATER	gavage	10	A8.5	cortical bone	normal	n	na	na	na	na	na	na	na
IBaCCHistoPath1	Hamster	8	F	IBACC	gavage	3	B1.5	cortical bone	normal	n	na	na	na	na	na	na	na
IBaCCHistoPath1	Hamster	8	F	IBACC	gavage	3	B2.5	cortical bone	normal	n	na	na	na	na	na	na	na
IBaCCHistoPath1	Hamster	8	F	WATER	gavage	3	B3.5	cortical bone	normal	n	na	na	na	na	na	na	na
IBaCCHistoPath1	Hamster	8	F	WATER	gavage	3	B4.5	cortical bone	normal	n	na	na	na	na	na	na	na
IBaCCHistoPath1	Hamster	8	M	IBACC	gavage	3	B5.5	cortical bone	normal	n	na	na	na	na	na	na	na
IBaCCHistoPath1	Hamster	8	M	IBACC	gavage	3	B6.5	cortical bone	normal	n	na	na	na	na	na	na	na
IBaCCHistoPath1	Hamster	8	M	WATER	gavage	3	B8.1	stomach, squamous	ingested material in mucus	y	incidental	na	diffuse	na	na	na	na
IBaCCHistoPath1	Hamster	8	M	IBACC	gavage	10	A1 .1	stomach, glandular	mucosal mineralized foci	y	mineralized foci	mild	mf	mucosa	periperal lymphocytes, epithelioid macs	y	na
IBaCCHistoPath1	Hamster	8	M	IBACC	gavage	10	A1 .1	SI	plasma cell infiltrates	n	na	minimal	mf	lamina propria	plasma cells	n	expected variation in resident immune cells
IBaCCHistoPath1	Hamster	8	F	IBACC	gavage	3	B1.1	Peyer's patches	normal	n	na	na	na	na	na	na	na
IBaCCHistoPath1	Hamster	8	F	IBACC	gavage	3	B1.1	pancreas	normal	n	na	na	na	na	na	na	na
IBaCCHistoPath1	Hamster	8	F	IBACC	gavage	3	B1.1	LI	normal	n	na	na	na	na	na	na	na
IBaCCHistoPath1	Hamster	8	M	WATER	gavage	10	A4.1	stomach, squamous	na	na	na	na	na	na	na	na	na
IBaCCHistoPath1	Hamster	8	M	IBACC	gavage	10	A2.1	stomach, glandular	mucosal mineralized foci	y	mineralized foci	mild	mf	mucosa	na	y	na
IBaCCHistoPath1	Hamster	8	M	IBACC	gavage	10	A2.1	SI	eosinophilic and lymphoplasmacytic infiltrates	n	na	mild	mf	lamina propria	eosinophils & plasma cells	n	expected variation in resident immune cells
IBaCCHistoPath1	Hamster	8	F	IBACC	gavage	3	B2.1	Peyer's patches	normal	n	na	na	na	na	na	na	na
IBaCCHistoPath1	Hamster	8	F	IBACC	gavage	3	B2.1	pancreas	normal	n	na	na	na	na	na	na	na
IBaCCHistoPath1	Hamster	8	F	IBACC	gavage	3	B2.1	LI	normal	n	na	na	na	na	na	na	na
IBaCCHistoPath1	Hamster	8	M	IBACC	gavage	10	A1.1	stomach, squamous	normal	n	na	na	na	na	na	na	na
IBaCCHistoPath1	Hamster	8	M	WATER	gavage	10	A3.1	stomach, glandular	mucosal mineralized foci	n	na	na	na	na	na	na	na
IBaCCHistoPath1	Hamster	8	M	WATER	gavage	10	A3.1	SI	plasmacytic infiltrates	n	na	moderate	mf	lamina propria	plasma cells	n	expected variation in resident immune cells
IBaCCHistoPath1	Hamster	8	F	WATER	gavage	3	B3.1	Peyer's patches	normal	n	na	na	na	na	na	na	na
IBaCCHistoPath1	Hamster	8	F	WATER	gavage	3	B3.1	pancreas	normal	n	na	na	na	na	na	na	na
IBaCCHistoPath1	Hamster	8	F	WATER	gavage	3	B3.1	LI	normal	n	na	na	na	na	na	na	na
IBaCCHistoPath1	Hamster	8	M	IBACC	gavage	10	A2.1	stomach, squamous	normal	n	na	na	na	na	na	na	na
IBaCCHistoPath1	Hamster	8	M	WATER	gavage	10	A4.1	stomach, glandular	ingested material in mucus	y	incidental	na	diffuse	mucus	na	na	na
IBaCCHistoPath1	Hamster	8	M	WATER	gavage	10	A4.1	SI	eosinophilic and lymphoplasmacytic infiltrtates	n	na	mild	mf	lamina propria	eosinophils & plasma cells	n	expected variation in resident immune cells
IBaCCHistoPath1	Hamster	8	F	WATER	gavage	3	B4.1	Peyer's patches	normal	n	na	na	na	na	na	na	na
IBaCCHistoPath1	Hamster	8	F	WATER	gavage	3	B4.1	pancreas	normal	n	na	na	na	na	na	na	na
IBaCCHistoPath1	Hamster	8	F	WATER	gavage	3	B4.1	LI	normal	n	na	na	na	na	na	na	na
IBaCCHistoPath1	Hamster	8	M	IBACC	gavage	3	B5.1	stomach, squamous	normal	n	na	na	na	na	na	na	na
IBaCCHistoPath1	Hamster	8	M	IBACC	gavage	3	B5.1	stomach, glandular	ingested material in mucus; mucosal mineralized foci	y	mineralized foci	mild	mf	mucus; mucosa	na	y	na
IBaCCHistoPath1	Hamster	8	M	IBACC	gavage	3	B5.1	SI	plasma cell infiltrates	n	na	minimal	mf	lamina propria	plasma cells	n	expected variation in resident immune cells
IBaCCHistoPath1	Hamster	8	M	IBACC	gavage	3	B5.1	Peyer's patches	normal	n	na	na	na	na	na	na	na
IBaCCHistoPath1	Hamster	8	M	IBACC	gavage	3	B5.1	pancreas	normal	n	na	na	na	na	na	na	na
IBaCCHistoPath1	Hamster	8	M	IBACC	gavage	3	B5.1	LI	normal	n	na	na	na	na	na	na	na
IBaCCHistoPath1	Hamster	8	M	IBACC	gavage	3	B6.1	stomach, squamous	normal	n	na	na	na	na	na	na	na
IBaCCHistoPath1	Hamster	8	M	IBACC	gavage	3	B6.1	stomach, glandular	mucosal mineralized foci	y	mineralized foci	minimal	mf	mucosa	na	y	na
IBaCCHistoPath1	Hamster	8	M	IBACC	gavage	3	B6.1	SI	normal	n	na	na	na	na	na	na	na
IBaCCHistoPath1	Hamster	8	M	IBACC	gavage	3	B6.1	Peyer's patches	normal	n	na	na	na	na	na	na	na
IBaCCHistoPath1	Hamster	8	M	IBACC	gavage	3	B6.1	pancreas	na	na	na	na	na	na	na	na	na
IBaCCHistoPath1	Hamster	8	M	IBACC	gavage	3	B6.1	LI	normal	n	na	na	na	na	na	na	na
IBaCCHistoPath1	Hamster	8	M	WATER	gavage	10	A3.1	stomach, squamous	normal	n	na	na	na	na	na	na	na
IBaCCHistoPath1	Hamster	8	M	WATER	gavage	3	B7.1	stomach, glandular	mucosal mineralized foci; multifocal mineralization of small arterioles	y	mineralized foci	mild	mf	mucosa; small arterioles in lamina propria/muscularis mucosa	endothelial cells & smooth muscle	y	na
IBaCCHistoPath1	Hamster	8	M	WATER	gavage	3	B7.1	SI	plasma cell infiltrates	n	na	minimal	mf	lamina propria	plasma cells	n	expected variation in resident immune cells
IBaCCHistoPath1	Hamster	8	M	WATER	gavage	3	B7.1	Peyer's patches	normal	n	na	na	na	na	na	na	na
IBaCCHistoPath1	Hamster	8	M	WATER	gavage	3	B7.1	pancreas	na	na	na	na	na	na	na	na	na
IBaCCHistoPath1	Hamster	8	M	WATER	gavage	3	B7.1	LI	normal	n	na	na	na	na	na	na	na
IBaCCHistoPath1	Hamster	8	M	WATER	gavage	3	B7.1	stomach, squamous	normal	n	na	na	na	na	na	na	na
IBaCCHistoPath1	Hamster	8	M	WATER	gavage	3	B8.1	stomach, glandular	single mineralized mucosal cell	y	mineralized foci	minimal	solitary	mucosa	epithelial cell	y	na
IBaCCHistoPath1	Hamster	8	M	WATER	gavage	3	B8.1	SI	normal	n	na	na	na	na	na	na	na
IBaCCHistoPath1	Hamster	8	M	WATER	gavage	3	B8.1	Peyer's patches	normal	n	na	na	na	na	na	na	na
IBaCCHistoPath1	Hamster	8	M	WATER	gavage	3	B8.1	pancreas	normal	n	na	na	na	na	na	na	na
IBaCCHistoPath1	Hamster	8	M	WATER	gavage	3	B8.1	LI	normal	n	na	na	na	na	na	na	na
IBaCCHistoPath1	Hamster	8	M	WATER	gavage	3	B7.5	cortical bone	normal	n	na	na	na	na	na	na	na
IBaCCHistoPath1	Hamster	8	M	IBACC	gavage	10	A2.1	kidney	mineralized foci	y	mineralized foci	mild	mf	collecting ducts	na	y	na
IBaCCHistoPath1	Hamster	8	M	WATER	gavage	3	B7.8	cortical bone	normal	n	na	na	na	na	na	na	na

IBaCCHistoPath1	Hamster	8	F	IBACC	gavage	3	B2.1	spleen	normal	n	na	na	na	na	na	na	na
IBaCCHistoPath1	Hamster	8	M	IBACC	gavage	10	A2.1	mesentery	lymphocytic serositis with mesothelial hypertrophy	y	inflammation, hypertrophy	moderate	diffuse	mesentery	lymphocytes, mesothelial cells	incidental	incidental
IBaCCHistoPath1	Hamster	8	M	IBACC	gavage	10	A1.5	growth plate	normal	n	na	na	na	na	na	na	na
IBaCCHistoPath1	Hamster	8	M	IBACC	gavage	10	A2.2	kidney	mineralized foci; tubular dilation, epithelial degeneration	y	mineralized foci; degeneration	mild	mf; focal	collecting ducts; cortex	na	incidental	na
IBaCCHistoPath1	Hamster	8	F	IBACC	gavage	3	B2.2	spleen	normal	n	na	na	na	na	na	na	na
IBaCCHistoPath1	Hamster	8	M	IBACC	gavage	10	A2.2	mesentery	lymphocytic serositis with mesothelial hypertrophy	y	inflammation, hypertrophy	mild	diffuse	mesentery	lymphocytes, mesothelial cells	incidental	incidental
IBaCCHistoPath1	Hamster	8	M	IBACC	gavage	10	A2.5	growth plate	normal	n	na	na	na	na	na	na	na
IBaCCHistoPath1	Hamster	8	M	WATER	gavage	10	A3.2	kidney	immature tubules	y	developmental	minimal	mf	cortex	epithelial cell	na	na
IBaCCHistoPath1	Hamster	8	M	WATER	gavage	10	A3.5	growth plate	normal	n	na	na	na	na	na	na	na
IBaCCHistoPath1	Hamster	8	F	WATER	gavage	3	B3.2	spleen	normal	n	na	na	na	na	na	na	na
IBaCCHistoPath1	Hamster	8	M	WATER	gavage	10	A3.2	mesentery	lymphocytic serositis with mesothelial hypertrophy	y	inflammation, hypertrophy	minimal	mf	mesentery	lymphocytes, mesothelial cells	incidental	incidental
IBaCCHistoPath1	Hamster	8	M	WATER	gavage	10	A4.5	growth plate	normal	n	na	na	na	na	na	na	na
IBaCCHistoPath1	Hamster	8	M	WATER	gavage	10	A4.2	kidney	mineralized foci	y	mineralized foci	minimal	mf	collecting ducts	na	y	na
IBaCCHistoPath1	Hamster	8	F	WATER	gavage	3	B4.2	spleen	normal	n	na	na	na	na	na	na	na
IBaCCHistoPath1	Hamster	8	M	WATER	gavage	10	A4.2	mesentery	lymphocytic serositis with mesothelial hypertrophy	y	inflammation, hypertrophy	mild	mf	mesentery	lymphocytes, mesothelial cells	incidental	incidental
IBaCCHistoPath1	Hamster	8	F	IBACC	gavage	10	A5.5	growth plate	normal	n	na	na	na	na	na	na	na
IBaCCHistoPath1	Hamster	8	M	IBACC	gavage	3	B5.2	kidney	mineralized foci	y	mineralized foci	mild	mf	cortical tubules/collecting ducts	epithelial cell	y	na
IBaCCHistoPath1	Hamster	8	M	IBACC	gavage	3	B5.2	spleen	normal	n	na	na	na	na	na	na	na
IBaCCHistoPath1	Hamster	8	M	IBACC	gavage	3	B5.2	mesentery	lymphocytic serositis with mesothelial hypertrophy	y	inflammation, hypertrophy	mild	diffuse	mesentery	lymphocytes, mesothelial cells	incidental	incidental
IBaCCHistoPath1	Hamster	8	F	IBACC	gavage	10	A6.5	growth plate	normal	n	na	na	na	na	na	na	na
IBaCCHistoPath1	Hamster	8	M	IBACC	gavage	3	B6.2	kidney	mineralized foci	y	mineralized foci	mild	mf	collecting ducts	epithelial cell	y	na
IBaCCHistoPath1	Hamster	8	M	IBACC	gavage	3	B6.2	spleen	normal	n	na	na	na	na	na	na	na
IBaCCHistoPath1	Hamster	8	M	IBACC	gavage	3	B6.2	mesentery	too small to eval.	na	na	na	na	na	na	na	na
IBaCCHistoPath1	Hamster	8	F	WATER	gavage	10	A7.5	growth plate	normal	n	na	na	na	na	na	na	na
IBaCCHistoPath1	Hamster	8	M	WATER	gavage	3	B7.2	kidney	mineralized foci	y	mineralized foci	minimal	mf	collecting ducts	epithelial cell	y	na
IBaCCHistoPath1	Hamster	8	M	WATER	gavage	3	B7.2	spleen	normal	n	na	na	na	na	na	na	na
IBaCCHistoPath1	Hamster	8	M	WATER	gavage	3	B7.2	mesentery	normal	n	na	na	na	na	na	na	na
IBaCCHistoPath1	Hamster	8	F	WATER	gavage	10	A8.5	growth plate	normal	n	na	na	na	na	na	na	na
IBaCCHistoPath1	Hamster	8	M	WATER	gavage	3	B8.2	kidney	mineralized foci	y	mineralized foci	mild	mf	collecting ducts	epithelial cell	y	na
IBaCCHistoPath1	Hamster	8	M	WATER	gavage	3	B8.2	spleen	normal	n	na	na	na	na	na	na	na
IBaCCHistoPath1	Hamster	8	M	WATER	gavage	3	B8.2	mesentery	normal	n	na	na	na	na	na	na	na
IBaCCHistoPath1	Hamster	8	F	IBACC	gavage	3	B1.5	growth plate	normal	n	na	na	na	na	na	na	na
IBaCCHistoPath1	Hamster	8	F	IBACC	gavage	3	B2.5	growth plate	normal	n	na	na	na	na	na	na	na
IBaCCHistoPath1	Hamster	8	F	WATER	gavage	3	B3.5	growth plate	normal	n	na	na	na	na	na	na	na
IBaCCHistoPath1	Hamster	8	F	WATER	gavage	3	B4.5	growth plate	normal	n	na	na	na	na	na	na	na
IBaCCHistoPath1	Hamster	8	M	IBACC	gavage	3	B5.5	growth plate	normal	n	na	na	na	na	na	na	na
IBaCCHistoPath1	Hamster	8	M	IBACC	gavage	3	B6.5	growth plate	normal	n	na	na	na	na	na	na	na
IBaCCHistoPath1	Hamster	8	M	WATER	gavage	3	B7.5	growth plate	normal	n	na	na	na	na	na	na	na
IBaCCHistoPath1	Hamster	8	M	WATER	gavage	3	B7.8	growth plate	normal	n	na	na	na	na	na	na	na
IBaCCHistoPath1	Hamster	8	F	IBACC	gavage	3	B1.4	lungs	normal	n	na	na	na	na	na	na	na
IBaCCHistoPath1	Hamster	8	M	IBACC	gavage	10	A1.4	heart	normal	n	na	na	na	na	na	na	na
IBaCCHistoPath1	Hamster	8	M	IBACC	gavage	10	A1.4	thymus	na	na	na	na	na	na	na	na	na
IBaCCHistoPath1	Hamster	8	F	IBACC	gavage	3	B2.4	lungs	normal	n	na	na	na	na	na	na	na
IBaCCHistoPath1	Hamster	8	M	IBACC	gavage	10	A2.4	heart	normal	n	na	na	na	na	na	na	na
IBaCCHistoPath1	Hamster	8	M	IBACC	gavage	10	A2.4	thymus	normal	n	na	na	na	na	na	na	na
IBaCCHistoPath1	Hamster	8	F	WATER	gavage	3	B3.4	lungs	normal	n	na	na	na	na	na	na	na
IBaCCHistoPath1	Hamster	8	M	WATER	gavage	10	A3.4	heart	normal	n	na	na	na	na	na	na	na
IBaCCHistoPath1	Hamster	8	M	WATER	gavage	10	A3.4	thymus	normal	n	na	na	na	na	na	na	na
IBaCCHistoPath1	Hamster	8	F	WATER	gavage	3	B4.4	lungs	normal	n	na	na	na	na	na	na	na
IBaCCHistoPath1	Hamster	8	M	WATER	gavage	10	A4.7	heart	normal	n	na	na	na	na	na	na	na
IBaCCHistoPath1	Hamster	8	M	WATER	gavage	10	A4.8	thymus	normal	n	na	na	na	na	na	na	na
IBaCCHistoPath1	Hamster	8	M	IBACC	gavage	3	B5.4	lungs	normal	n	na	na	na	na	na	na	na
IBaCCHistoPath1	Hamster	8	F	IBACC	gavage	10	A5.4	heart	normal	n	na	na	na	na	na	na	na
IBaCCHistoPath1	Hamster	8	M	IBACC	gavage	3	B5.4	thymus	normal	n	na	na	na	na	na	na	na
IBaCCHistoPath1	Hamster	8	M	IBACC	gavage	3	B6.4	lungs	normal	n	na	na	na	na	na	na	na
IBaCCHistoPath1	Hamster	8	F	IBACC	gavage	10	A6.4	heart	normal	n	na	na	na	na	na	na	na
IBaCCHistoPath1	Hamster	8	M	IBACC	gavage	3	B6.4	thymus	normal	n	na	na	na	na	na	na	na
IBaCCHistoPath1	Hamster	8	M	WATER	gavage	3	B7.4	lungs	normal	n	na	na	na	na	na	na	na
IBaCCHistoPath1	Hamster	8	F	WATER	gavage	10	A7.4	heart	normal	n	na	na	na	na	na	na	na
IBaCCHistoPath1	Hamster	8	M	WATER	gavage	3	B7.4	thymus	normal	n	na	na	na	na	na	na	na
IBaCCHistoPath1	Hamster	8	M	WATER	gavage	3	B8.4	lungs	normal	n	na	na	na	na	na	na	na
IBaCCHistoPath1	Hamster	8	F	WATER	gavage	10	A8.4	heart	normal	n	na	na	na	na	na	na	na
IBaCCHistoPath1	Hamster	8	M	WATER	gavage	3	B8.4	thymus	normal	n	na	na	na	na	na	na	na
IBaCCHistoPath1	Hamster	8	F	IBACC	gavage	3	B1.4	heart	normal	n	na	na	na	na	na	na	na
IBaCCHistoPath1	Hamster	8	F	IBACC	gavage	3	B2.4	heart	normal	n	na	na	na	na	na	na	na
IBaCCHistoPath1	Hamster	8	F	WATER	gavage	3	B3.4	heart	normal	n	na	na	na	na	na	na	na
IBaCCHistoPath1	Hamster	8	F	WATER	gavage	3	B4.4	heart	normal	n	na	na	na	na	na	na	na
IBaCCHistoPath1	Hamster	8	M	IBACC	gavage	3	B5.4	heart	normal	n	na	na	na	na	na	na	na
IBaCCHistoPath1	Hamster	8	M	IBACC	gavage	3	B6.4	heart	normal	n	na	na	na	na	na	na	na
IBaCCHistoPath1	Hamster	8	M	WATER	gavage	3	B7.4	heart	normal	n	na	na	na	na	na	na	na
IBaCCHistoPath1	Hamster	8	M	WATER	gavage	3	B8.4	heart	normal	n	na	na	na	na	na	na	na
IBaCCHistoPath1	Hamster	8	M	IBACC	gavage	10	A2.1	liver	normal	n	na	na	na	na	na	na	na
IBaCCHistoPath1	Hamster	8	M	IBACC	gavage	10	A2.2	liver	normal	n	na	na	na	na	na	na	na
IBaCCHistoPath1	Hamster	8	M	WATER	gavage	10	A3.2	liver	normal	n	na	na	na	na	na	na	na
IBaCCHistoPath1	Hamster	8	M	WATER	gavage	10	A4.2	liver	normal	n	na	na	na	na	na	na	na
IBaCCHistoPath1	Hamster	8	F	IBACC	gavage	10	A5.2	liver	normal	n	na	na	na	na	na	na	na
IBaCCHistoPath1	Hamster	8	F	IBACC	gavage	10	A6.2	liver	normal	n	na	na	na	na	na	na	na
IBaCCHistoPath1	Hamster	8	F	WATER	gavage	10	A7.2	liver	normal	n	na	na	na	na	na	na	na
IBaCCHistoPath1	Hamster	8	F	WATER	gavage	10	A8.2	liver	normal	n	na	na	na	na	na	na	na
IBaCCHistoPath1	Hamster	8	F	IBACC	gavage	3	B2.1	liver	normal	n	na	na	na	na	na	na	na
IBaCCHistoPath1	Hamster	8	F	IBACC	gavage	3	B2.2	liver	normal	n	na	na	na	na	na	na	na
IBaCCHistoPath1	Hamster	8	F	IBACC	gavage	3	B2.2	liver	normal	n	na	na	na	na	na	na	na
IBaCCHistoPath1	Hamster	8	F	WATER	gavage	3	B4.2	liver	normal	n	na	na	na	na	na	na	na
IBaCCHistoPath1	Hamster	8	M	IBACC	gavage	3	B5.2	liver	normal	n	na	na	na	na	na	na	na
IBaCCHistoPath1	Hamster	8	M	IBACC	gavage	3	B6.2	liver	normal	n	na	na	na	na	na	na	na
IBaCCHistoPath1	Hamster	8	M	WATER	gavage	3	B7.2	liver	normal	n	na	na	na	na	na	na	na
IBaCCHistoPath1	Hamster	8	M	WATER	gavage	3	B8.2	liver	normal	n	na	na	na	na	na	na	na

Table S4. Serum biochemistry values for all animals in histopathology study. Missing values are due to limited amount of serum available for particular animals.
P values are two-tailed Mann-Whitney (MW; http://www.statskingdom.com/170median_mann_whitney.html) comparing all Cry5B IBaCC-treated hamsters versus all Water-treated hamsters for any given parameter.
Further breakdown (based on gender, days post last gavage or days post) are not possible due to limited number of animals per group. Only average sodium levels were statistically different (slightly elevated 142 mEq/L in IBaCC vs 138 mEq/L in Water).
Columns in color are relevant for determining any potential hepatic and renal injury. NA = no statistical comparison possible due to nature readout but clearly no difference between IBaCC and Water groups is seen.

**treatment**	**gende**	**days pos**	**Glucose mg/dL**	**Urea mg/dL**	**Creatinin mg/dL**	**Phosphoru mg/dL**	**Calcium 2 mg/dL**	**Magnesiu mg/dL**	**Total prote g/dL**	**Albumin g/dL**	**Globulins g/dL**	**A/G ratio**	**Sodium mEq/L**	**Chloride mEq/L**	**Potassium mEq/L**	**tCO2(Bicar mEq/L**	**AGAP**	**NA/K**	**total bi mg/dL**	**Alkaline Ph U/L**	**GGT U/L**	**ALT U/L**	**AST U/L**	**Creatine U/L**	**Choles mg/dL**	**Triglyce mg/dL**	**Amyla U/L**	**Osmolarity mmol/L**
IBaCC	M	10	126		0.2	15.5	11.3	5.4		3.7			146	92	8.4			17		163	<1	79	50	487	117	360	2869	
IBaCC	M	10	500		0.4	25.6	12.3	7.1	6.2	3.7	2.5	1.5	138	85	14			10		173	<1	161	183	2077	108	459	2620	
IBaCC	F	10	182	23	0.2	16.7	13.8	5.7	6.9	3.7	3.2	1.2	144	89	11.4	25	30	13		235	1	57	156	1653	110	306	2488	307
IBaCC	F	10			0.2		16.1	6.7		3.8			142	88	16.2			9		239	1	55	52	586	108	255	2548	
IBaCC	F	3	334	24	0.2	14.5	15.7	6.7	6.3	3.6	2.7	1.3	143	90	14	27	26	10	<0.1	225	<1	57	43	725	108	265	5758	319
IBaCC	F	3	389	22	0.2	16.3	16.3	7.1	6.4	3.6	2.8	1.3	142	88	16.5	27	27	9	<0.1	226	<1	57	35	387	115	264	6638	324
IBaCC	M	3					14	5.4		3.5			141	91	14.3			10		173		82		1986	115			
IBaCC	M	3	488	25	0.3	16.2	15.7	6.8	6.6	3.9	2.7	1.4	142	91	17	18	33	8	<0.1	138	<1	69	119	1117	125	291	4454	332
Water	M	10	573	25	0.4	17.1	13	7	5.9	3.5	2.4	1.5	131	80	9.8	16	35	13		152	<1	283	161	755	107	349	4826	303
Water	M	10	500		0.2	16.8	13.3	7		3.7			133	83	18.1			7		176	<1	98	88	646	102	346	3303	
Water	F	10	274	21	0.2	16.9	15.4	6.1	6.1	3.5	2.6	1.3	141	88	13.5	28	25	10	<1	243	<1	56	55	654	111	274	6289	310
Water	F	10			0.2		14.6	6.7		3.6			136	84	21.2			6		226	<1	318		903	96	318		
Water	F	3	458	21	0.2	20.2	16.8	7.2	6.5	3.7	2.8	1.3	139	89	21.2	25	25	7	<0.1	241	<1	68	70	626	106	267	6610	331
Water	F	3	349	23	0.3	18.3	14.6	5.9	6.1	3.5	2.6	1.3	145	93	9.6	26	26	15	<0.1	198	<1	86	84	451	71	138	5407	315
Water	M	3	490	26	0.2	15	15.6	7.2	6.5	3.9	2.6	1.5	139	87	16.8	25	27	8	<0.1	187	2	82	45	370	111	272	5664	326
Water	M	3	388	22	0.3	16.8	12.1	6	6.5	3.9	2.6	1.5	141	89	18.6	19	33	8	<0.1	154	<1	68	204	1744	132	430	3336	326
**P value (MW)**			**0.32**	**0.67**	**0.84**	**0.17**	**0.79**	**0.37**	**0.31**	**0.67**	**0.19**	**0.43**	**0.039**	**0.15**	**0.27**	**0.75**	**0.67**	**0.18**	**NA**	**0.75**	**NA**	**0.17**	**0.46**	**0.44**	**0.1**	**0.87**	0.21	0.91
